# Molecular mechanisms underlying the structural diversity of rhamnose-rich cell wall polysaccharides in lactococci

**DOI:** 10.1016/j.jbc.2023.105578

**Published:** 2023-12-16

**Authors:** Hugo Guérin, Pascal Courtin, Alain Guillot, Christine Péchoux, Jennifer Mahony, Douwe van Sinderen, Saulius Kulakauskas, Christian Cambillau, Thierry Touzé, Marie-Pierre Chapot-Chartier

**Affiliations:** 1 Université Paris-Saclay, INRAE, AgroParisTech, Micalis Institute, Jouy-en-Josas, France; 2 Université Paris-Saclay INRAE, AgroParisTech, GABI, Jouy-en-Josas, France; 3 School of Microbiology and APC Microbiome Ireland, University College Cork, Cork, Ireland; 4 Laboratoire d’Ingénierie des Systèmes Macromoléculaires (LISM), Institut de Microbiologie, Bioénergies et Biotechnologie (IMM), Aix-Marseille Université – CNRS, UMR 7255, Marseille, France; 5 Université Paris-Saclay, CEA, CNRS, Institute for Integrative Biology of the Cell (I2BC), Gif-sur-Yvette, France

**Keywords:** cell wall, polysaccharide, glycosyltransferase, gram-positive bacteria, bacteriophage, *Lactococcus*

## Abstract

In Gram-positive bacteria, cell wall polysaccharides (CWPS) play critical roles in bacterial cell wall homeostasis and bacterial interactions with their immediate surroundings. In lactococci, CWPS consist of two components: a conserved rhamnan embedded in the peptidoglycan layer and a surface-exposed polysaccharide pellicle (PSP), which are linked together to form a large rhamnose-rich CWPS (Rha-CWPS). PSP, whose structure varies from strain to strain, is a receptor for many bacteriophages infecting lactococci. Here, we examined the first two steps of PSP biosynthesis, using *in vitro* enzymatic tests with lipid acceptor substrates combined with LC-MS analysis, AlfaFold2 modeling of protein 3D-structure, complementation experiments, and phage assays. We show that the PSP repeat unit is assembled on an undecaprenyl-monophosphate (C_55_P) lipid intermediate. Synthesis is initiated by the WpsA/WpsB complex with GlcNAc-P-C_55_ synthase activity and the PSP precursor GlcNAc-P-C_55_ is then elongated by specific glycosyltransferases that vary among lactococcal strains, resulting in PSPs with diverse structures. Also, we engineered the PSP biosynthesis pathway in lactococci to obtain a chimeric PSP structure, confirming the predicted glycosyltransferase specificities. This enabled us to highlight the importance of a single sugar residue of the PSP repeat unit in phage recognition. In conclusion, our results support a novel pathway for PSP biosynthesis on a lipid-monophosphate intermediate as an extracellular modification of rhamnan, unveiling an assembly machinery for complex Rha-CWPS with structural diversity in lactococci.

The Gram-positive bacteria cell wall consists of an intricate and highly organized assembly of glycopolymers and proteins. It is composed of a thick layer of peptidoglycan ensuring bacterial cell shape and mechanical resistance, which is functionalized with so-called secondary cell wall glycopolymers that perform multiple functions (1, 2). These secondary cell wall glycopolymers include wall teichoic acids (WTA), well characterized in numerous Firmicutes (3), and rhamnose-rich polysaccharides (Rha-CWPS) present in certain ovoid-shaped Firmicutes lacking (or producing a low amount of) WTA, such as pathogenic streptococci and enterococci or dairy streptococci and lactococci (4, 5). Rha-CWPS are composed of a polyrhamnose core (also named rhamnan) with side-chain substituents of variable size and structure according to the species or strain (4). Like WTAs, Rha-CWPS appear as anionic glycopolymers covalently bound to peptidoglycan and may represent half the cell wall mass. In recent years, they emerged as functional analogs of WTAs, with a crucial role in bacterial growth and division, and in the interplay of bacteria with their environment including interactions with infecting bacteriophages or host cells (4, 5, 6).

*Lactococcus lactis* and *Lactococcus cremoris* are lactic acid bacteria widely used as starter cultures in dairy fermentations. As such, they suffer bacteriophage infections that may cause fermentation failures and economic losses (7, 8). For the majority of lactococcal phages belonging to the *Caudoviridetes* class, Rha-CWPS are recognized by the receptor-binding protein (RBP) located at the phage tail tip, allowing adsorption of phage particles at the bacterial surface (7, 9). In lactococci, Rha-CWPS are complex heteropolysaccharides comprised of a rhamnan backbone chain and side-chain substituents made of oligosaccharide or polysaccharide chains with variable structures between strains (10, 11). The variable part of Rha-CWPS constitutes the phage receptor, which at least in part explains the narrow host range of lactococcal phages (12, 13). The Rha-CWPS structural diversity has been linked to genetic diversity in the *cwps* gene cluster, which was used as a basis to classify *L. lactis/L. cremoris* strains in four groups (A to D) (11). The *cwps* gene cluster consists of a conserved 5′-region responsible for rhamnan backbone synthesis, and a more variable 3′-region responsible for the synthesis of variable side-chain substituents (14). In C-type strains, the variable moiety of Rha-CWPS is a polysaccharide detected as an outer layer at the bacterial surface and previously named polysaccharide pellicle (PSP) (12, 15, 16), whereas it is an oligosaccharide in A- and B-type strains (17, 18). We have previously proposed a lactococcal Rha-CWPS biosynthesis scheme, in which rhamnan and PSP are independently synthesized and then covalently linked at the extracellular side of the cytoplasmic membrane (Fig. 1) (14). In this model, PSP is presumed to constitute an extracellular modification of rhamnan, synthesized on an undecaprenyl-monophosphate (C_55_P) lipid intermediate and transferred by a GT-C fold glycosyltransferase (19, 20) onto the rhamnan chain. Synthesis of the PSP repeat unit is hypothesized to be initiated by the glycosyltransferase WpsA (with WpsB, a small membrane protein, as an activator) by transferring a GlcNAc residue from UDP-GlcNAc onto C_55_P thus generating GlcNAc-P-C_55_. The repeat unit is then assembled on the GlcNAc-P-C_55_ lipid intermediate by sequential addition of several sugars (typically four in *L. cremoris* NZ9000) by dedicated glycosyltransferases at the inner side of the cytoplasmic membrane. The linear oligosaccharide repeat unit linked to C_55_P is then flipped towards the outer side of the membrane by flippase WpsG. In C-type strains, the PSP repeat unit is likely transferred to the growing PSP chain by the GT-C fold glycosyltransferase WpsH, resulting in its polymerization, with WpsI as a putative chain-length regulator. Finally, PSP is transferred from C_55_P onto rhamnan by the GT-C fold glycosyltransferase, WpsJ, and the C_55_P can then be recycled (21, 22).

**Figure 1:**
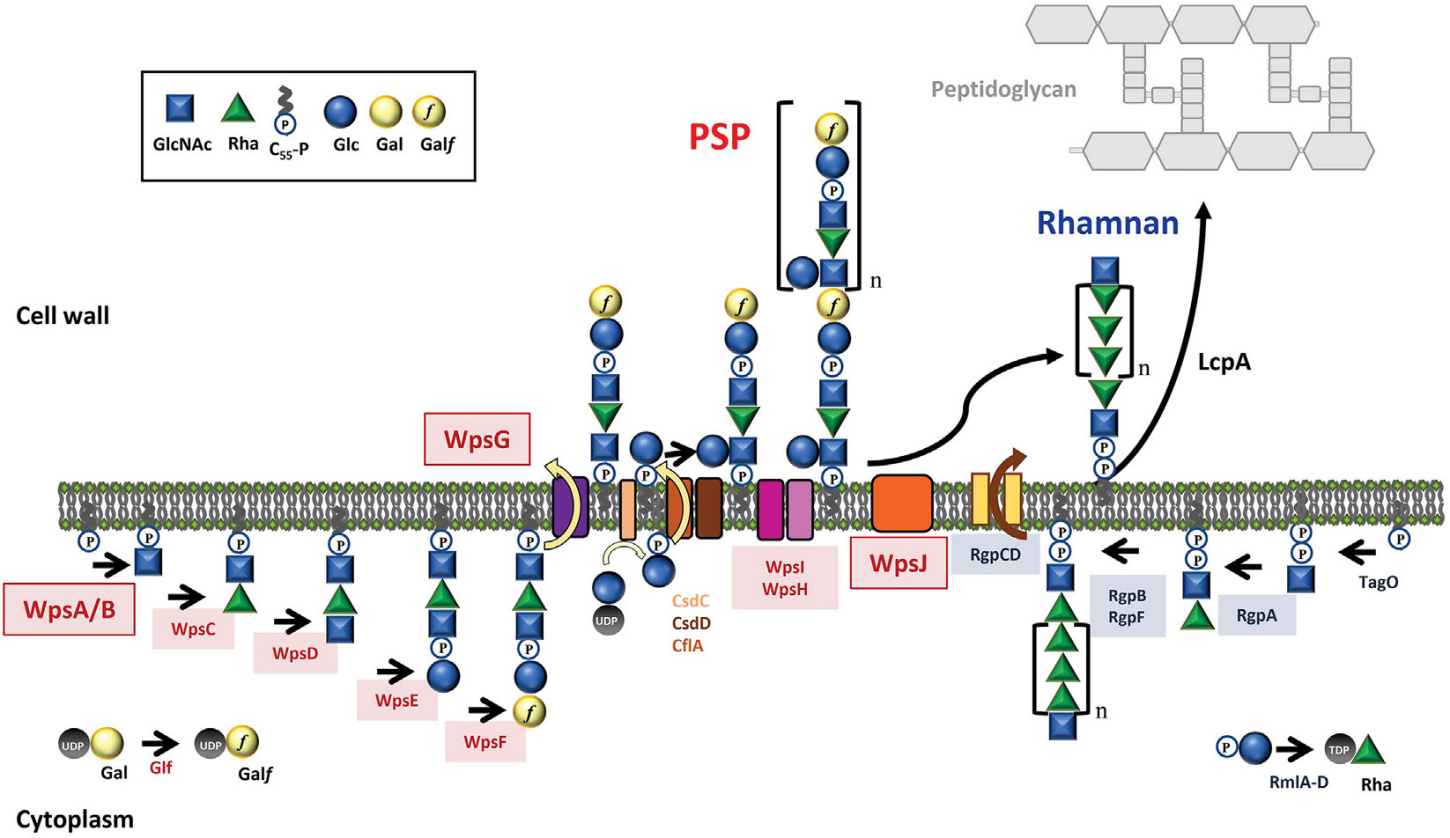
**Model scheme for rhamnan and PSP biosynthesis in *Lactococcus cremoris* NZ9000.** (*Right side*) Rhamnan biosynthesis is initiated by the transfer of GlcNAc-P on C_55_P by TagO, yielding GlcNAc-PP-C_55_. The nascent chain is then elongated by the addition of Rha residues, from the dTDP-Rha donor synthesized by RmlABCD, by RgpB and RgpF, and possibly terminated by the transfer of a GlcNAc residue by RgpE. The polyrhamnose chain is exported through the membrane by the ABC transporter RgpCD and anchored to the cell wall, presumably on a MurNAc (or GlcNAc) residue of the glycan chains of peptidoglycan by LcpA. (*Left side*) PSP biosynthesis is initiated by the transfer of a GlcNAc residue on C_55_P by WpsA activated by WpsB, yielding GlcNAc-P-C_55_. The repeat unit is elongated by the processive addition of Rha, GlcNAc, Glc-P, and Gal*f* (from UDP-Gal*f* produced by Glf) residues by glycosyltransferases WpsC, WpsD, WpsE, and WpsF respectively and exported to the extracytoplasmic side of the membrane by the Wzx-like flippase, WspG. The lateral Glc residue of the PSP repeat unit is added outside the cytoplasm by the three-component glycosylation system CsdC/CsdD/CflA (36), encoded by genes not belonging to the *cwps* cluster. The PSP repeat units are polymerized by WpsI and its regulator WpsH. PSP is anchored to the rhamnan chain by WpsJ. This model was initially proposed in (14).

The model proposed for PSP biosynthesis was based on bioinformatic analysis and gene inactivation (14). In the current study, we aimed at validating experimentally the first enzymatic steps of PSP biosynthesis by *in vitro* activity tests and *in vivo* complementation experiments. We demonstrated the activity of the WpsA/WpsB complex in the initiation of PSP biosynthesis by transferring a GlcNAc residue on C_55_P. We also highlighted the activity of a second glycosyltransferase, which is specific to a given bacterial strain, in the elongation of the oligosaccharide repeat unit on GlcNAc-P-C_55_, thereby demonstrating that PSP synthesis occurs on a C_55_P lipid carrier, with a monophosphate linkage between the lipid tail and the PSP repeat unit, and illustrating the generation of PSP structural diversity. Furthermore, based on the predicted specificities of glycosyltransferases involved in PSP repeat unit elongation in two different strains, we engineered a PSP biosynthesis pathway to obtain a modified PSP structure and further examined its impact on phage infection.

## Results

### Detection of GlcNAc-P-C_55_ synthesis activity in *L. cremoris* membranes

**Figure 2:**
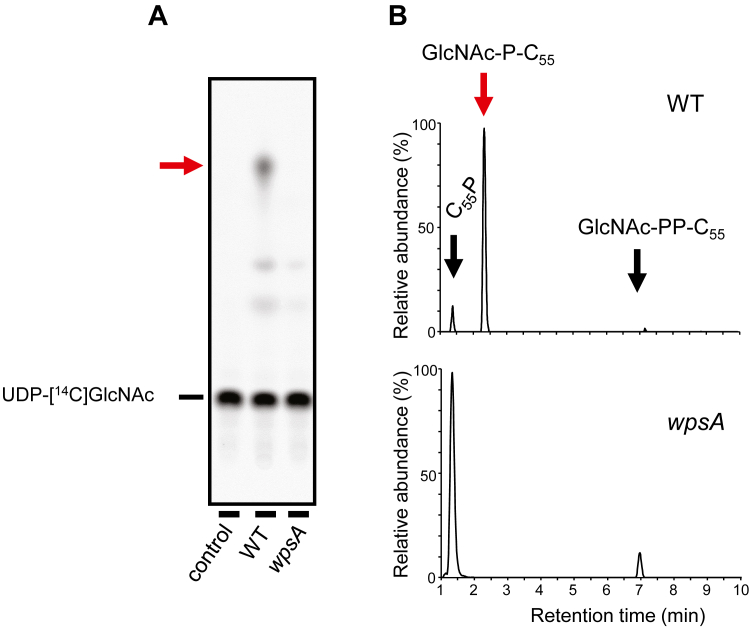
**Detection of GlcNAc-P-C**_**55**_**synthesis activity in membranes from *Lactococcus cremoris* WT and *wpsA* mutant.***A*, TLC analysis of the reaction mixtures revealed by radioactivity detection. The first lane (control) corresponds to reaction mixture without membranes. *Red arrow* indicates the newly formed product containing [^14^C]GlcNAc. *B*, LC-MS analysis in the negative ion mode of butanol-extracted C_55_P derivatives. Combined extracted ion chromatograms (EICs) for C_55_P, GlcNAc-P-C_55_, and GlcNAc-PP-C_55_ ions ([M-H]^-^).

We first tested the ability of wild-type (WT) *L. cremoris* NZ9000 strain and NZ9000 *wpsA* mutant to synthesize GlcNAc-P-C_55_ *in vitro*, by examining membrane fractions of these strains, which were incubated with radiolabeled UDP-[^14^C]GlcNAc as a donor substrate and C_55_P as an acceptor substrate. The reaction mixtures were analyzed by thin-layer chromatography (TLC) on a silica plate. A new radiolabeled species with higher mobility than UDP-[^14^C]GlcNAc and presumably more hydrophobic, was visualized in the presence of WT membranes but was absent with NZ9000 *wpsA* membranes (Fig. 2*A*).

The same reactions were performed with non-radioactive UDP-GlcNAc. C_55_P substrate and its derivative lipid products were extracted with butanol and analyzed by high-resolution LC-MS in negative-ion mode (Fig. 2*B*). In both WT and NZ9000 *wpsA* strains, a molecular ion with an m/z value of 845.659 [M-H]^-^ was detected in agreement with the calculated mass of the C_55_P substrate (Table 1). In addition, a molecular ion with an m/z value of 1048.736 [M-H]^-^ consistent with the calculated mass of GlcNAc-P-C_55_ (Table 1) was detected in the WT sample, but was absent in the *wpsA* mutant. By comparison, a molecular ion with an m/z value of 1128.703 [M-H]^-^ corresponding to the calculated mass of GlcNAc-PP-C_55_ (Table 1), expected to be the product of TagO involved in the initiation of rhamnan synthesis (see Fig. 1) (10), was detected in both WT and *wpsA* samples. Together, these results indicate the presence of a WpsA-dependent UDP-GlcNAc:C_55_P GlcNAc-transferase activity in *L. cremoris* NZ9000.

**Table 1:** Calculated and measured masses of C_55_P and its derivatives

Compound	Calculated mass	Calculated [M-H]^-^	Measured [M-H]^-^
C_55_P	846.666	845.658	845.659
GlcNAc-P-C_55_	1049.742	1048.734	1048.736
GlcNAc-PP-C_55_	1129.708	1128.700	1128.703
Rha-GlcNAc-P-C_55_	1195.803	1194.795	1194.783
Gal-GlcNAc-P-C_55_	1211.798	1210.790	1210.770

### WpsA is a GlcNAc-P-C_55_ synthase activated by WpsB

**Figure 3:**
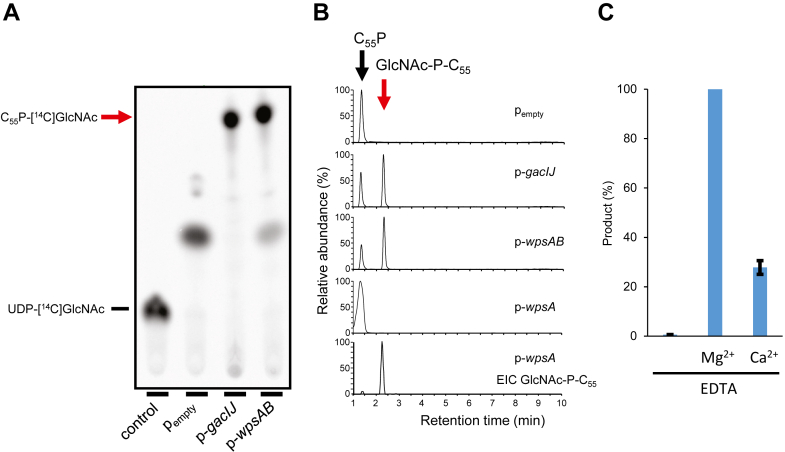
**Activity of WpsA and WpsB produced in *Escherichia coli* membranes.***A*, TLC analysis of the reaction mixtures revealed by radioactivity detection. The first lane (control) corresponds to reaction mixture without membranes. Membranes from *Escherichia coli* expressing GacI and GacJ were taken as positive control and membranes with empty plasmid pET21His30 as negative control. *Red* arrow indicates the newly formed product containing [^14^C]GlcNAc. *B*, LC-MS analysis in the negative ion mode of butanol-extracted C_55_P derivatives. Combined EICs for C_55_P and C_55_P-GlcNAc ions ([M-H]^-^) ions are shown for membranes from *E. coli* containing empty plasmid (p_empty_), p-*gacIJ*, p-*wpsAB* and p-*wpsA*, or EIC of the sole GlcNAc-P-C_55_P ion from *E. coli* (p-*wpsA*) membranes (bottom chromatogram). *C*, relative product formation in *E. coli* (p-*wpsAB*) membranes in the presence of 10 mM EDTA supplemented or not with 50 mM Mg^2+^ or Ca^2+^ cations. Product formation is expressed as a percentage of the radioactivity count associated with GlcNAc-P-C_55_P detected in the presence of Mg^2+^. Mean of three experiments.

Lactococcal WpsA and WpsB are homologs of *Streptococcus pyogenes* GacI and GacJ, sharing 43.8 and 33.3 % sequence identity, respectively. GacI was shown to possess UDP-GlcNAc:C_55_P GlcNAc transferase activity, while GacJ, a small membrane protein, forms a complex with GacI thereby enhancing its activity (23). To further investigate the role of WpsA and WpsB in GlcNAc-P-C_55_ synthesis, we expressed *wpsA* and *wpsB* in their original genetic organization (*i.e.* as a bicistronic transcriptional unit) or *wpsA* alone in *Escherichia coli*. In both cases, the recombinant proteins were associated with the membrane fraction (Fig. S1*A*). Membrane fractions containing WpsA, WpsA and WpsB, or GacI and GacJ (taken as a control) were tested for their ability to transfer GlcNAc from UDP-GlcNAc onto C_55_P. TLC analysis of the reaction mixtures (Figure 3, *A*) showed a major newly formed [^14^C]-labeled molecule in the presence of WpsA and WpsB, which was absent with the control membranes of *E. coli* carrying an empty plasmid vector. This radiolabeled molecule exhibited a similar TLC mobility as the product of GacI and GacJ (Fig. 3*A*), supporting our hypothesis that WpsA and WpsB catalyze GlcNAc-P-C_55_ formation.

LC-MS analysis of the extracted lipids from non-radioactive reaction mixtures detected a molecular ion with an m/z value of 1048.735 [M-H]^-^ in the presence of membrane fractions containing WpsA/WpsB (Fig. 3*B*), consistent with the calculated mass of GlcNAc-P-C_55_ (Table 1). This molecular ion was also present with the GacI/GacJ-containing membranes but not with membranes of *E. coli* carrying an empty plasmid. GlcNAc-P-C_55_ was also detected with membranes containing WpsA alone, though in a significantly lower abundance (Fig. 3*B*) thus indicating a positive effect of WpsB on WpsA activity.

### WpsA activity is dependent on divalent metal cations

WpsA belongs to the CAZy glycosyltransferase family 2 (GT2), predicted to adopt the well-described GT-A fold (24). Most glycosyltransferases with a GT-A fold contain a conserved DXD motif shown to coordinate Mg^2+^ or other metal divalent cations mediating the interaction with the nucleotide-sugar donor (19). WpsA sequence contains such a motif in positions 96 to 98. To assess the importance of divalent cations for WpsA activity, the *in vitro* enzymatic assay with *E. coli* membrane expressing WpsA/WpsB was performed as described above in the presence of EDTA. TLC analysis revealed that the presence of EDTA abolished GlcNAc-P-C_55_ synthesis, which in turn was restored upon the addition of MgCl_2_ (Fig. 3*C*). Synthesis of GlcNAc-P-C_55_ was only partially restored (four-fold reduction in product formation) by the addition of the same concentration of CaCl_2_.

### Structural model of the WpsA/WpsB complex

**Figure 4:**
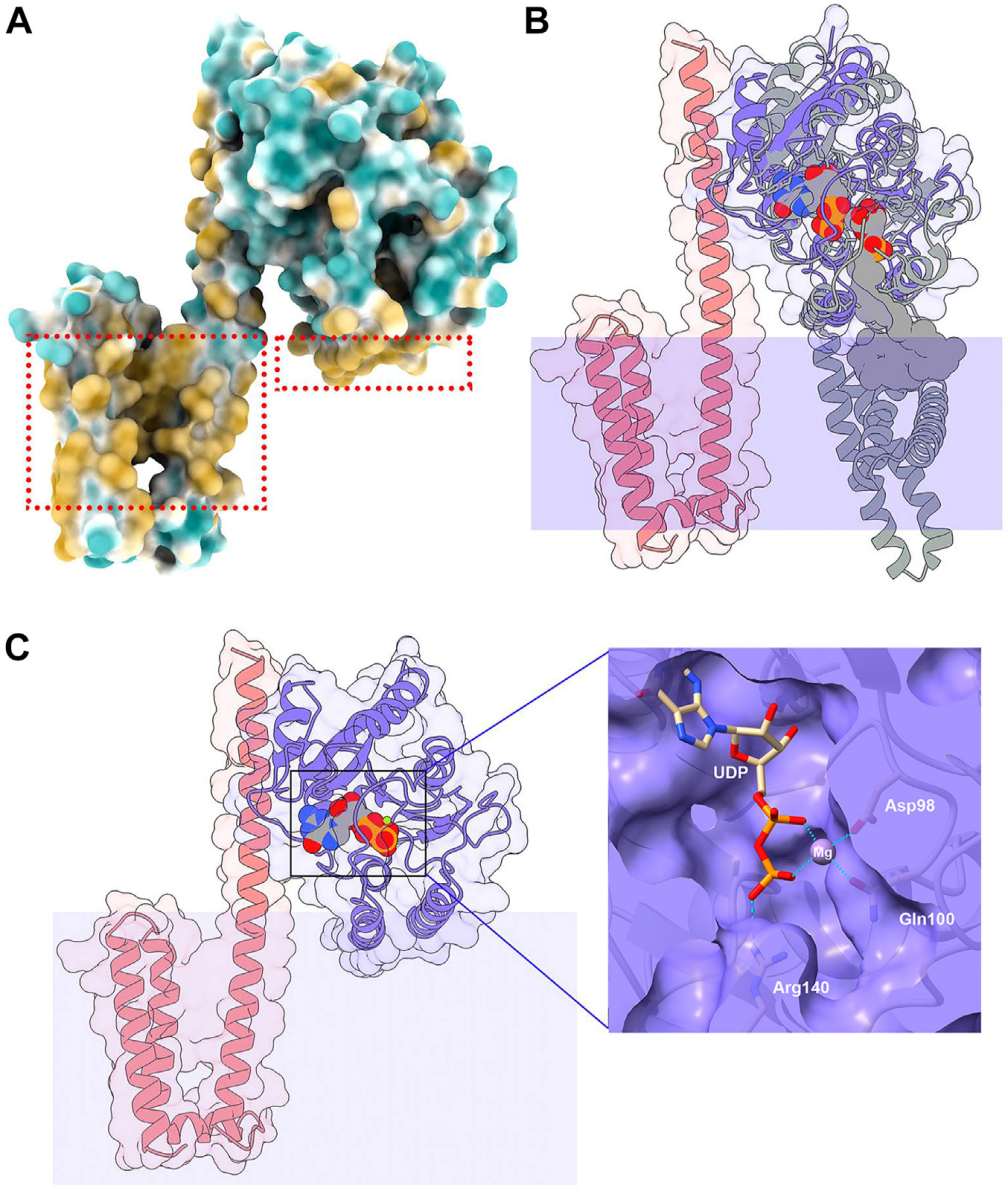
**The WpsA/WpsB complex modeled with Alphafold2**. *A*, the surface of the complex is colored according to hydrophilicity (*cyan*) and hydrophobicity (*orange*). The hydrophobic surfaces of WpsB (*left*) and WpsA (*right*) are boxed. *B*, Ribbon view and surface of superposed WpsA/B and dolichyl phosphate mannose synthase (DPMS) from *Pyrococcus furiosus* (PDB ID 5mm1). WpsB and WpsA are colored *magenta* and *violet*, respectively. DPMS is *gray*, and UDP and dolichyl phosphate mannose are represented. The cytoplasmic membrane position is suggested by a *violet rectangle*. *C*, Ribbon view and transparent surface of WpsA/B with UDP and Mg^2+^ arising from superposition with DPMS GDP complex (PDB ID 5mlz). *Inset*: View of UDP and Mg^2+^ binding site, involving Asp98 and Gln100 (Mg^2+^) and Arg 140 (PO_4_ of UDP).

*S. pyogenes* GacI and GacJ form a detergent-stable and active complex (23). To evaluate WpsA/WpsB complex formation, WpsA and WpsB 3D structures were modeled with AlphaFold2. The program predicted a complex between WpsA and WpsB with good pLDDT values (Fig. S2). WpsA is formed of a 7-stranded central β-sheet in which six β-strands are parallel and one antiparallel (Fig. 4). Three α-helices cover one face of the β-sheet and five α-helices cover the other side. WpsB is formed of four α-helices (Fig. 4). The first two helices form a helical hairpin and are followed by a short helix perpendicular to the hairpin. The fourth long helix of WpsB is roughly parallel to the helical hairpin and contacts WpsA at its N-terminus. Interactions between the two proteins involve residues 58 to 87 in WpsA and 93 to 115 in WpsB. WpsA/WpsB interaction analysis by PISA (25) reveals that 635 Å^2^ and 741 Å^2^ of WpsA and WpsB surfaces are buried in the interaction, respectively (Fig. S3). TMHMM (26) predicted three transmembrane helices in WpsB, and none in WpsA (Fig. S4). However, a hydrophobic bump between WpsA residues ∼215 to 235 suggests that membrane-associated helices may exist (Fig. S4). This was confirmed using ChimeraX (27) hydrophobic patches prediction that shows that the two C-terminal helices of WpsA display a hydrophobic face (Fig. 4*A*), which could be exposed towards the membrane. Consequently, WpsA is most probably linked to the inner side of the cytoplasmic membrane through complex formation with WpsB and direct interaction of its C-terminal amphipathic helices with the membrane (Fig. 4*A*). Moreover, according to the predicted structures, WpsA/WpsB complex formation allows the suitable positioning of the WpsA catalytic site towards its membrane-embedded lipid acceptor substrate.

**Figure 5:**
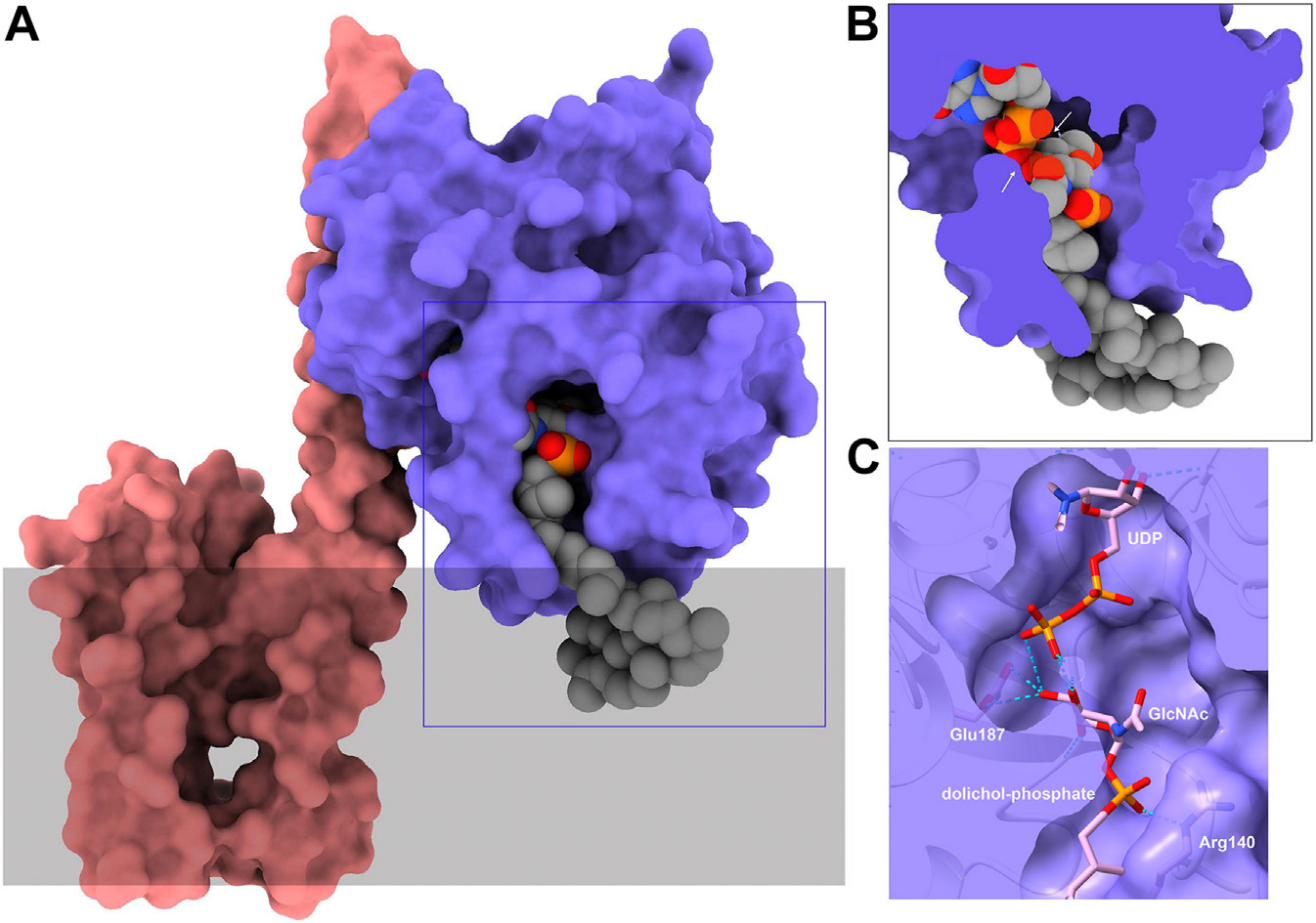
**The WpsA/WpsB complex with ligands.** The surface of the complex is colored *magenta* (WpsB) and *blue* (WpsA). UDP and dolichyl phosphate GlcNAc (DPGN) are colored *gray* (carbon), *red* (oxygen), *blue* (nitrogen), and *orange* (phosphate). *A*, UDP and a part of DPGN are hidden by a bridge over the active site, with two doors on the *left* for UDP-GlcNAc and on the *right* for dolichyl phosphate. *B*, the active site has been slabbed revealing the ligands. The two arrows show the contact area between UDP and DPGN. *C*, Residues at the vicinity of ligands are Arg140 that binds to the phosphate of DPM and Glu187 that contacts the GlcNAc product and may be involved in catalysis.

We further performed a Dali (28) search of WpsA, which returned several excellent hits (Fig. S5), the best ones involving a dolichol monophosphate mannose synthase (DPMS) from the archaea, *Pyrococcus furiosus.* DPMS catalyzes the transfer of mannose (Man) from GDP-mannose to the dolichylphosphate (Dol-P) carrier, to yield Dol-P-Man and is involved in protein glycosylation. Three high-resolution crystal structures of DPMS are available, in complex with GDP nucleotide (PDB ID 5mlz, Z = 24.4, r.m.sd = 2.6 Å), GDP-Man donor (PDB ID 5mm0), and Dol-P-Man glycolipid product (PDB ID 5mm1) (29). Superposition of WpsA and 5mm1 structure reveals that in 5mm1, the transmembrane helices display a different position than WpsB helices, but are on the same face of the catalytic domain (Fig. 4*B*). Superposition of the UDP-complexed DPMS (5mlz) reveals that WpsA possesses the same catalytic machinery (Fig. 4*C* and inset). In the WpsA model structure, Arg140 binds the distal phosphate moiety of UDP and Asp98 and Gln100 C=O moiety bind to the Mg^2+^ ion, vs Asp91 and Gln93 in *Pyroccocus* DPMS (29). The side-chain of Asp96 that is part of the DXD motif of WpsA, is close to the Mg^2+^ ion but not at a binding distance = d=5.6 Å), a feature also present in DPMS (Asp89). When superimposed to DPMS UDP and Dol-P-Man complex (5mm1), we noticed that, provided a small translation-rotation, the Dol-P chain could fit well in a WpsA crevice and a generated model of Dol-P-β-GlcNAc (Fig. 5, *A* and *B*). The phosphate moiety of the Dol-P-β-GlcNAc is bound to Arg140, and GlcNAc interacts with Glu187 that may be part of the catalytic machinery (Fig. 5*C*). WpsA belongs to the CAZy GT2 family and is consequently presumed to be an inverting GT-A fold glycosyltransferase, in which the catalytic mechanism is an S_N_2-like reaction, requiring a base catalyst (Glu or Asp) for the nucleophilic attack on the acceptor substrate (19). In WpsA, Glu187 is thus a possible candidate to fulfill this function.

### Expression of an active WpsBA fusion protein

Attempts to purify the WpsA/WpsB complex with various detergents to assess its enzymatic activity were unsuccessful. According to the structure prediction of the WpsA/WpsB complex, the WpsB C-terminal end is located close to the WpsA N-terminal end. We thus tested the activity of a fusion protein (named WpsBA^fu^) where the C-terminus of WpsB is fused to the N-terminus of WpsA. WpsBA^fu^ was produced in *E. coli* (Fig. S1*B*) and the corresponding membrane fraction was tested *in vitro* for GlcNAc-P-C_55_ synthesis activity as described above. TLC analysis revealed the synthesis of a new radiolabeled product with the same mobility as the one produced by membranes expressing WpsA and WpsB (Fig. S6*A*). Moreover, with purified WpsBA^fu^ protein (Fig. S1*C*), the same product was observed, although with low intensity (Fig. S6*A*`). LC-MS analysis confirmed the presence of GlcNAc-P-C_55_ in both membrane-bound and purified WpsBA^fu^ reaction mixtures (Fig. S6*B*). In contrast, a similar experiment performed with *E. coli* membranes expressing a fusion protein (called WpsAB^fu^) comprising WpsA at the N-terminus and WpsB at the C-terminus revealed no activity (data not shown). All these results support the predicted WpsA/B complex model and confirm that WpsA and WpsB form a functional membrane-associated complex.

### Strain-dependent elongation of the PSP repeat unit

**Figure 6:**
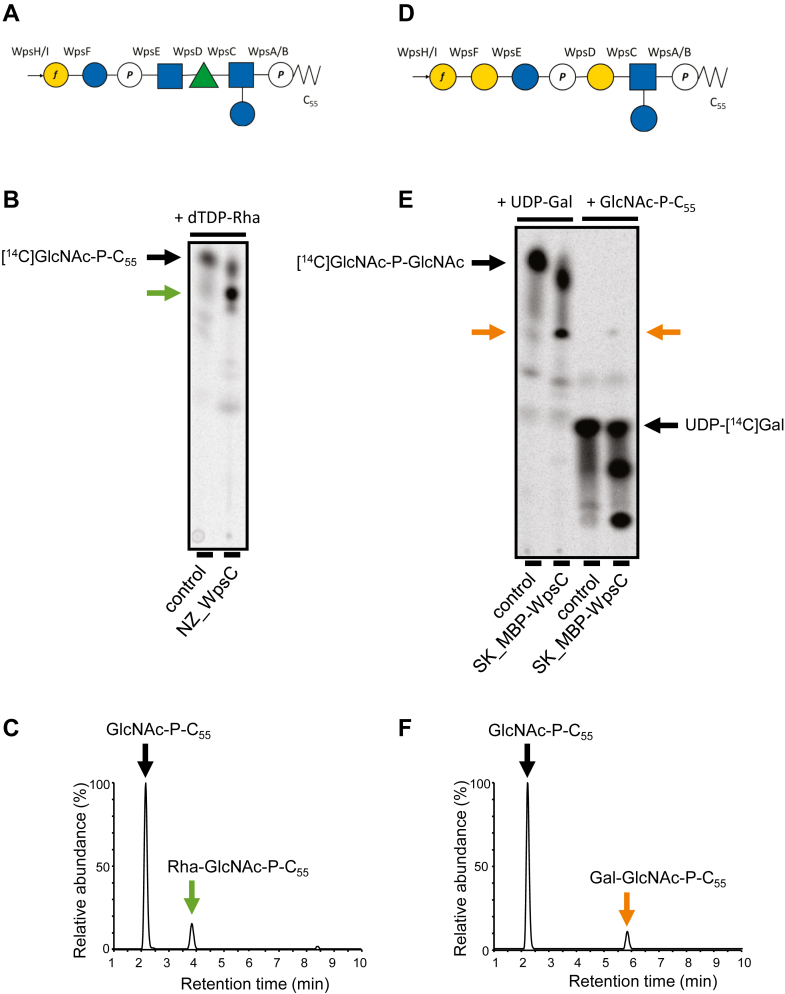
**Activity detection of NZ_WpsC and MBP-SK_WpsC.***A* and *D*, structure of the PSP repeat unit of *L. cremoris* NZ9000 (*A*) and SK11 (*D*) (11, 15). Proteins proposed to be involved in the synthesis of each glycosidic linkage are indicated (see also Fig. 1). *Blue* square, GlcNAc; *green* triangle, Rha; *blue* circle, Glc; *yellow* circle, Gal; *f*, furanose; P, phosphate. *B*, TLC analysis of the reaction mixtures containing purified NZ_WpsC, [^14^C]GlcNAc-P-C_55_ acceptor substrate and TDP-Rha donor substrate, revealed by radioactivity detection. The *green* arrow indicates the newly formed product containing [^14^C]GlcNAc. *C*, LC-MS analysis in the negative ion mode of butanol-extracted C_55_P derivatives. Combined EICs for GlcNAc-P-C_55_ and Rha-GlcNAc-P-C_55_ ([M-H]^-^) ions are shown. *E*, TLC analysis of the reaction mixtures containing purified MBP-SK_WpsC, [^14^C]GlcNAc-P-C_55_ acceptor substrate and UDP-Gal donor substrate, or GlcNAc-P-C_55_ acceptor substrate and UDP-[^14^C]Gal donor substrate revealed by radioactivity detection. The *orange* arrows indicate the newly formed product containing [^14^C]GlcNAc (*left*) or [^14^C]Gal (*right*). *F*, LC-MS analysis in the negative ion mode of butanol-extracted C_55_P derivatives. Combined EICs for GlcNAc-P-C_55_ and Gal-GlcNAc-P-C_55_ ([M-H]^-^) ions are shown.

Following the addition of a GlcNAc residue onto C_55_P, the nascent PSP repeat unit is proposed to be elongated at the membrane cytoplasmic side by the sequential action of glycosyltransferases using specific sugar-nucleotide substrates (Fig. 1). The large diversity of the lactococcal PSP structures parallels the diversity in glycosyltransferase-encoding genes in the *cwps* locus across *L. lactis/L. cremoris* strains (11, 14). Whereas WpsA and WpsB are highly conserved between lactococcal strains, the glycosyltransferase encoded by the gene (named *wpsC*) located downstream of *wpsA* and *wpsB* is not conserved between C-type strains. We investigated the enzymatic activity of WpsC of 2 *L. cremoris* strains, NZ9000 and SK11, producing PSP with different structures (Fig. 6, *A* and *D*). These enzymes, called NZ-WpsC and SK_WpsC, respectively, exhibit no sequence similarity and were predicted to catalyze the addition of a Rha residue and a galactose (Gal) residue, respectively, on the GlcNAc-P-C_55_ glycolipid intermediate (Fig. 6, *A* and *D*).

In most bacteria, the nucleotide sugar donor of rhamnosyltransferases involved in polysaccharide biosynthesis is dTDP-Rha (30, 31, 32). Purified recombinant NZ_WpsC (Fig. S1*D*) was incubated with radiolabeled [^14^C]GlcNAc-P-C_55_ and dTDP-Rha. TLC analysis showed a novel radiolabeled molecule in the presence of NZ_WpsC with a reduced migration distance as compared to GlcNAc-P-C_55_, suggesting a more hydrophilic compound, as expected for Rha-GlcNAc-P-C_55_ (Fig. 6*B*). LC-MS analysis of the extracted lipids from an identical non-radioactive reaction mixture detected a molecular ion with m/z value of 1194.783 [M-H]^-^ in the presence of NZ_WpsC (Fig. 6*C*), consistent with the calculated mass of Rha-GlcNAc-P-C_55_ (Table 1).

SK_WpsC was produced in *E. coli* as a fusion protein with an N-terminal maltose-binding protein (MBP) tag. Purified MBP-SK_WpsC (Fig. S1*E*) was incubated with [^14^C]GlcNAc-P-C_55_ and UDP-Gal. A novel radiolabeled molecular species was detected by TLC analysis with a reduced migration distance as compared to GlcNAc-P-C_55_, indicating the formation of a more hydrophilic product (Fig. 6*E*). Also, a radiolabeled molecule with the same mobility was detected when MBP-SK_WpsC was incubated with GlcNAc-P-C_55_ and radiolabeled UDP-[^14^C]Gal (Fig. 6*E*). Together, these results suggest that SK_WpsC transfers a Gal residue from UDP-Gal onto GlcNAc-P-C_55_. This was confirmed by high-resolution negative-ion LC-MS analysis of the lipids extracted from a reaction mixture with non-radiolabeled substrates (Fig. 6*F*). A molecular ion with an m/z value of 1210.770 [M-H]^-^ was detected in the presence of MBP-SK_WpsC, consistent with the calculated mass of a Gal-GlcNAc-P-C_55_ (Table 1).

In conclusion, both glycosyltransferases, NZ_WpsC and SK_WpsC, are active on the GlcNAc-P-C_55_ acceptor substrate and can elongate the chain on the C_55_-monophosphate intermediate with the predicted enzymatic specificities. These results validate the first steps of our proposed model of PSP biosynthesis.

### Engineering a chimeric PSP structure

**Figure 7:**
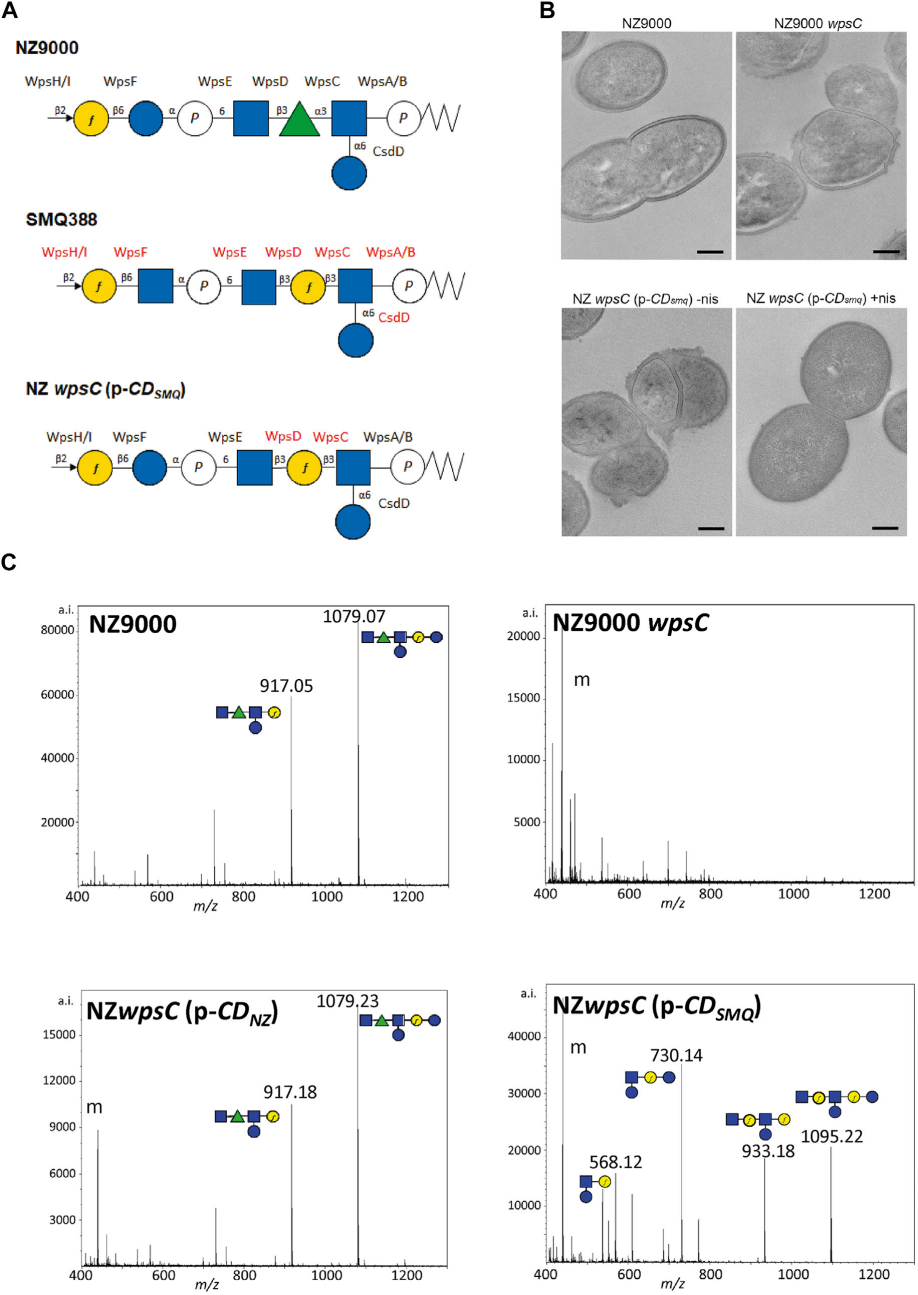
**Construction of a chimeric PSP.***A*, structure of the PSP repeat unit linked to C_55_P from *L. cremoris* NZ9000 and SMQ-388 and expected structure for the mutant strain *wpsC* (p-*CD*_*SMQ*_). NZ9000 and SMQ-388 PSP structures were established previously by NMR analysis (15, 16). The proteins proposed to be involved in the formation of each glycosidic linkage are indicated (14). *Blue* square, GlcNAc; *green* triangle, Rha; *blue* circle, Glc; *yellow* circle, Gal; *f,* furanose; P, phosphate. *B*, TEM micrographs of *L. cremoris* NZ9000, NZ9000 *wpsC*, and NZ *wpsC* (p-*CD*_*SMQ*_) cultured without (-nis) or with nisin (+nis). The electron-dense outer layer visible in WT NZ9000 cells was previously attributed to the PSP (15). Scale bars represent 200 nm. *C*, MALDI-TOF mass spectra of the purified PSP oligosaccharides from *L. cremoris* NZ9000 and mutant derivatives. *m/z* values correspond to [M+Na]^+^ adducts. *L. cremoris* NZ9000 synthesizes polymeric PSP that depolymerized during HF extraction by cleavage at the level of phosphodiester bonds leading to hexasaccharide (calculated *m/z* [M+Na]^+^1079.37). HF also partially cleaves between Gal*f* and Glc leading to a pentasaccharide fragment (calculated *m/z* [M+Na]^+^ 917.32), as shown previously by NMR analysis (15). An identical spectrum is observed for control strain *wpsC* (p-*CD*_*NZ*_) PSP. HF cleavages are also observed in *wpsC* (p-*CD*_*SMQ*_) PSP, leading to fragments with calculated *m/z* ([M+Na]^+^) values of 1095.37, 933.32, 730.24, and 568.18. m, matrix peaks. *Blue* square, GlcNAc; *green* triangle, Rha; *blue* circle, Glc; *yellow* circle, Gal; *f*, furanose.

Glycosyltransferase activities are highly specific for their donor and acceptor substrates (19). By comparing the previously elucidated PSP structures of *L. cremoris* NZ9000 and SMQ-388 (15, 16), we observed that glycosyltransferases NZ_WpsC and SMQ_WpsC are proposed to transfer a different sugar moiety onto the same acceptor GlcNAc-P-C_55_ whereas, at the next step, the nascent PSP repeat unit is proposed to be elongated by the same sugar (GlcNAc) in these two strains (Fig. 7*A*). As depicted in Figure 7*A* and as shown above, NZ_WpsC catalyzes the transfer of a Rha residue onto GlcNAc-P-C_55_ to form Rha-GlcNAc-P-C_55_. The lipid-linked-disaccharide is then proposed to be elongated by the addition of a GlcNAc by NZ_WpsD, thus yielding GlcNAc-Rha-GlcNAc-P-C_55_. In strain SMQ-388, WpsC and WpsD are proposed to transfer a galactofuranose (Gal*f*) and then a GlcNAc residue, respectively, leading to GlcNAc-Gal*f*-GlcNAc-P-C_55_ lipid intermediate (Fig. 7*A*). To obtain a chimeric PSP between NZ9000 and SMQ-388 (*i.e.* to replace the Rha residue by a Gal*f* residue in NZ9000 PSP), we complemented the mutant NZ9000 *wpsC* with *wpsC* and *wpsD* genes from SMQ-388, placed under the control of a nisin-inducible promoter (strain NZ *wpsC* (p-*CD*_*SMQ*_)). As a control, genes *wpsC* and *wpsD* from NZ9000 were expressed in NZ9000 *wpsC* under the nisin inducible promoter (strain NZ *wpsC* (p-*CD*_*NZ*_)). Similarly, with the same reasoning, a second recombinant strain was constructed to obtain a chimeric PSP (with a GlcNAc-P replacing the Glc-P), by cloning genes *wpsE* and *wpsF* from SMQ-388 in NZ9000 *wpsE* mutant (NZ *wpsE* (p-*EF*_*SMQ*_)) with the respective control (NZ *wpsE* (p-*EF*_*NZ*_)).

We analyzed the CWPS content of the complemented strains grown in the presence of nisin. CWPS were released from cell walls by hydrofluoric acid (HF) treatment that cleaves the phosphate bond between rhamnan and peptidoglycan and depolymerizes PSP by cleavage of the phosphodiester bonds inside the PSP chain (10). Rhamnan and PSP oligosaccharides were then separated by size-exclusion high-pressure liquid chromatography (SEC-HPLC) (Fig. S7). The same profile as that of WT NZ9000 with rhamnan and PSP oligosaccharide peaks, was observed for the complemented strain NZ *wpsC* (p-*CD*_*NZ*_). In the NZ9000 *wpsC* mutant, the PSP peak is lacking whereas in the complemented strain NZ *wpsC* (p-*CD*_*SMQ*_), it was greatly reduced. Fractions collected at the level of the PSP peak were further analyzed by Maldi-TOF MS (Fig. 7*C*). As described previously (14), the spectrum obtained for WT NZ9000 PSP reveals two main peaks at m/z of 1079.07 assigned to the hexasaccharide resulting from PSP chain depolymerization by HF cleavage at phosphodiester bonds and at m/z 917.05 assigned to the pentasaccharide obtained by partial cleavage of the hexasaccharide by HF after Gal*f* residues (Fig. 7*C*). A similar spectrum was obtained for control NZ *wpsC* (p-*CD*_*NZ*_) PSP. In contrast, the spectrum obtained for the PSP of NZ *wpsC* (p-*CD*_*SMQ*_) was different. It contained four major peaks with m/z values of 1095.22, 933.18, 730.14, and 568.12 [M+Na]^+^, which correspond to the calculated masses of the expected modified PSP hexasaccharide repeat unit (with a Gal*f* residue replacing the Rha residue of NZ9000) and of degradation products resulting from partial cleavage by HF after Gal*f* residues (Fig. 7*C*). In addition, the detected molecular ions confirmed the polymeric nature of the chimeric PSP, since they would not be found in a single repeat unit monomer (Fig. 7*A*). Together, these results validate the enzymatic specificities assigned to NZ_WpsC and SMQ_WpsC glycosyltransferases and support our PSP biosynthesis model.

When the same CWPS analysis was performed with NZ *wpsE* (p-*EF*_*SMQ*_), only rhamnan but no PSP was detected in the CWPS extract analyzed by SEC-HPLC (data not shown) and no chimeric PSP could be detected by Maldi-TOF MS analysis (Fig. S8).

We examined the morphology of the complemented strains by transmission electron microscopy (TEM). NZ9000 *wpsC* mutant exhibits the typical defects reported previously for PSP-negative lactococcal cells including loss of the ovoid shape and alteration of the cell division process leading to aggregates (14, 15, 33) (Fig. 7*B*). Also, the electron-dense outer layer ascribed to PSP observed in NZ9000 is no longer visible. The same phenotype was observed for the *wpsC* (p-*CD*_*SMQ*_) strain in the absence of nisin (Fig. 7*B*). Upon nisin addition, as demonstrated above, the modified PSP is produced, although at a substantially lower level than in WT NZ9000, which could account for barely visible PSP on TEM micrographs (Fig. 7*B*). Nevertheless, cell shape was restored and most aggregates disappeared (Fig. 7*B*), confirming that expression of SMQ-388 glycosyltransferases WpsC and WpsD in NZ9000 *wpsC* mutant allows PSP synthesis. Thus, although the modified PSP is produced in a markedly lower amount as compared to the WT one, it is sufficient to substantially alleviate the morphological and division defects of NZ9000 *wpsC* mutant.

### Impact of a single monosaccharide change in the PSP repeat unit on phage sensitivity

To evaluate the effect of Rha to Gal*f* substitution in NZ9000 PSP on phage sensitivity, the NZ *wpsC* (p-*CD*_*SMQ*_) strain induced by nisin was tested for its sensitivity to phages sk1 and p2. These phages are known to infect *L. cremoris* NZ9000 but not SMQ-388 and to recognize PSP as a receptor (15, 34). Previously, NZ9000 *wpsC*, devoid of PSP, was shown to be resistant to these phages (14). Upon induction with nisin, NZ *wpsC* (p-*CD*_*SMQ*_) remained immune to both tested phages (Table 2). Since NZ *wpsC* (p-*CD*_*SMQ*_) produced PSP in a lower amount as compared to the WT, we also tested NZ9000 *wpsB* mutant in the same phage assay, as a low PSP producer control. According to our previous study (14), NZ9000 *wpsB* produces native polymeric PSP, but in a much lower amount than the WT strain, as does the NZ *wpsC* (p-*CD*_*SMQ*_) strain (Fig. S7). However, contrary to NZ *wpsC* (p-*CD*_*SMQ*_), NZ9000 *wpsB* was still sensitive to sk1 and p2 with an efficiency of plaquing (E.O.P.) similar to that of NZ9000 (Table 2). Together, our results strongly suggest that the single exchange of Rha to Gal*f* in the PSP repeat unit confers resistance to phages sk1 and p2, most probably through the failure of the phage adsorption step.

**Table 2:** Sensitivity of *L. lactis* NZ9000, NZ9000 *wpsB*, SMQ-388, and *wpsC* (p-*CD_SMQ_*) to phages sk1 and p2

E.O.P. values^a^^,^^b^		
	sk1	p2

NZ9000	1	1
*wpsB*	8.8 ± 1.7 × 10^−1^	8.2 ± 1.5 × 10^−1^
SMQ-388	≤4.5 × 10^-9^	≤1.0 × 10^−10^
*wpsC* (p-*CD*_*SMQ*_)^c^	≤4.5 × 10^−9^	≤1.0 × 10^−10^

a E.O.P. values were calculated as the ratio of the phage titer on the tested strain by the phage titer on NZ9000. Values are the mean of three independent experiments.

b Phages and strains are listed in Table S1.

c Strain *wpsC* (p-*CD*_*SMQ*_) was grown in the presence of nisin.

## Discussion

Rha-CWPS are crucial cell wall components of several Gram-positive ovoid-shaped bacteria, with a role in cell wall biogenesis and cell division. They are engaged in multiple and crucial interactions of these bacteria with their surrounding medium. In most species, they are bacteriophage receptors and, in pathogenic streptococci, they have an established role as virulence factors (4). The side chain substituents of the rhamnan backbone chains, variable between species and even between strains within a species, are major determinants of Rha-CWPS functions. In lactococci, depending on the strain, rhamnan substituents comprised either polymeric chains (previously termed PSP) or oligosaccharidic chains. We have previously proposed a model for CWPS biosynthesis in lactococci with rhamnan and PSP being synthesized independently from two distinct lipid sugar precursors and linked after their transfer outside of the cytoplasmic membrane (14). In this study, we reconstituted the first cytoplasmic steps of the PSP biosynthesis scheme. Our results support the model where PSP is synthesized on a C_55_-monophosphate lipid intermediate, in line with PSP being an extracellular glycosidic modification of rhamnan.

Multicomponent transmembrane glycosylation systems, composed of three or four proteins, play a major role in the extracellular modification of diverse bacterial glycopolymers including lipopolysaccharides, WTAs, lipoteichoic acids and Rha-CWPS (4, 20, 35). In particular, *S. pyogenes* GacI and GacJ are thought to be part of a four-component glycosylation system, together with a Wzx-type flippase GacK and a GT-C fold glycosyltransferase GacL, involved in GlcNAc monosaccharide decoration of the rhamnan chain of group A carbohydrate (GAC) (23, 35). GacI (activated by GacJ) synthesizes GlcNAc-P-C_55_ at the inner side of the membrane, which is then flipped outside the membrane by GacK and GlcNAc is transferred by the GT-C fold enzyme GacL onto rhamnan with GlcNAc-P-C_55_ as donor substrate. We have shown in this study that lactococcal WpsA and WpsB, homologs of *S. pyogenes* GacI and GacJ, exhibit an identical enzymatic activity consisting in the catalysis of GlcNAc-P-C_55_ synthesis. However, in lactococci, as shown also here, GlcNAc-P-C_55_ is the acceptor substrate of cytoplasmic glycosyltransferases (WpsC) that catalyze the elongation of the PSP oligosaccharide repeat unit, by adding a specific sugar according to the bacterial strain. WpsA and WpsB can be considered as part of a four-component glycosylation system, comprising also a Wzx-type flippase (WpsG) and a GT-C fold glycosyltransferase (WpsJ) that would add the final PSP onto rhamnan (Fig. 1). In lactococci, this system is combined with other components including a series of intracellular glycosyltransferases to elongate the oligosaccharide repeat unit (up to five sugars) and proteins involved in repeat unit polymerization (WpsH and WpsI), to yield a sophisticated modification system dedicated to the synthesis and anchoring of polymeric PSP onto rhamnan (4, 14). Notably, the WpsA/B, Wzx-type flippase and GT-C fold glycosyltransferase WpsJ components are encoded in the genome of all known lactococcal strains, thus underlining the conservation of this modification scheme of rhamnan.

GacI and GacJ form a functional complex (23), but no 3D structure of the complex was available. Here, we modelled the protein structure of the WpsA/WpsB complex with AlphaFold2. Although WpsA has a lipid acceptor substrate, it is not an integral membrane protein, with no predicted TM segment. From the 3D model, we infer that WpsA association to the cytoplasmic membrane results from both its interaction with WpsB, which is an integral membrane protein with 3 TM segments, and the presence of amphipathic helices at the WpsA C-terminus. Therefore, the formation of the WpsA/WpsB complex positions the WpsA active site in the right orientation with respect to its membrane-embedded lipid substrate. The fact that a fusion protein WpsBA - but not a WpsAB fusion - is active, supports the predicted model for the WpsA/WpsB complex. Interestingly, and as observed previously (23), in certain bacterial species (including *Geobacter* sp., *Desulfuromonas soudanensis* or *Desulfurivibrio alkaliphilus*), homologs of GacJ and GacI are encoded as a fusion protein, with GacJ at the N-terminus (Fig. S9). With regard to WpsC and the other cytoplasmic glycosyltransferases that elongate the PSP repeat unit, it is tempting to speculate that they form a large protein complex with WpsA/WpsB. However, no complex could be predicted by Alphafold2 between WpsA/WpsB and WpsC.

Structural homology was found between lactococcal WpsA and DPMS that catalyzes the formation of a Dol-P-Man glycolipid intermediate, further used as an activated substrate for protein glycosylation in Archaea. Both WpsA and DPMS belong to the GT2 family of inverting glycosyltransferases with a GT-A fold. The superposition of the AlphaFold-modelled WpsA structure to the UDP-complexed DPMS crystal structure highlighted the conservation of the catalytic sites. In particular, both proteins possess a DXD motif that was shown previously in GT-A fold enzymes, to coordinate metal divalent ions required for catalysis, through correct positioning of the diphosphate group of NDP in the donor substrate (19). However, in the WpsA model as previously highlighted in the DPMS structure (29), Asp and Gln residues located in an extended Dx**D**x**Q** motif, bind to the metal divalent ion and thus coordinate the diphosphate group of the donor substrate (UDP or GDP), whereas the first Asp of the motif is not at a binding distance. Homology search and sequence alignment indicate that the DxDxQ motif is conserved in a number of enzymes of the GT2 family, which are polyisoprenyl-phosphate glycosyltransferases (including WpsA, DPMS, GacI and GtrB) (Fig. S10) that transfer a sugar from an NDP-sugar donor to a polyisoprenyl-phosphate acceptor substrate to generate lipid-monophosphate-sugar product. The DxDxQ motif could thus be a hallmark of this enzyme group inside the GT2 family.

The PSP structural diversity is, at least partly, responsible for the narrow host range of lactococcal phages. We observed that changing a single sugar (Rha to Gal*f*) of the NZ9000 PSP repeat unit abolished sk1 and p2 phage sensitivity. This is probably the consequence of a defect in phage adsorption at the surface of NZ9000-derived bacteria with a modified PSP. These results are in good agreement with a previously proposed model of RBP/PSP recognition specificity (34). As noted, *L. cremoris* strains NZ9000, SMQ-388 as well as a third strain, 3107, synthesize PSP made of phosphohexa- or pentasaccharide repeats sharing a semi-conserved trisaccharide motif (GlcNAc-Galf-GlcNAc-1P or GlcNAc-Galf-GlcNAc-1P) (Fig. S11) but they exhibit different sensitivity to phages (12, 16, 34). To explain the fine specificity of PSP recognition by phages, on the basis of X-ray crystallography data and docking approach, the model proposed that the trisaccharide motif could initiate binding of phage RBPs to PSP, while the more variable part of PSP would provide the binding specificity (34). The Rha residue is located in the variable part of the NZ9000 PSP repeat unit in agreement with a prevailing role in defining phage specificity.

Overall, our results emphasize the role of multicomponent transmembrane glycosylation systems in generating structural diversity of Rha-CWPS in *L. cremoris* and *L. lactis*. In addition, other three-component glycosylation systems involved in grafting side-chain Glc residues onto rhamnan and PSP have been previously described (36). The result is a high structural diversity of lactococcal Rha-CWPS between strains, which modulates their susceptibility to bacteriophages (11, 12). On the other hand, we have previously shown that Rha-CWPS, including both rhamnan and PSP components, are required for cell wall homeostasis and play a crucial role in bacterial cell morphogenesis and division (10, 33). In particular, the absence of the variable part of Rha-CWPS, the PSP, such as in a *wpsA* mutant, leads to strong morphological defects and alterations of septum positioning during cell division (33). This critical function of PSP could be linked to its negatively charged character, which is a conserved feature of the structurally variable substituents of rhamnan (4). Further studies are required to decipher at the molecular level how Rha-CWPS interact with the cell division machinery.

## Experimental procedures

### Bacterial strains, plasmids, bacteriophages and growth conditions

Bacterial strains, plasmids and bacteriophages used in this study are listed Table S1. *E. coli* strains were grown in Luria-Bertani (LB) broth (Difco) with aeration (200 rpm) at 37 °C unless otherwise stated. When required, 100 μg/ml of ampicillin (Amp) or 10 μg/ml of chloramphenicol (Cm) were added. *L. cremoris* strains were grown at 30 °C in M17 broth (Difco) supplemented with 0.5% (wt/vol) glucose (GM17). When required, 5 μg/ml Cm was added. To induce gene expression under the control of the *nisA* promoter, bacteria were precultured without nisin, and nisin A (Sigma-Aldrich) was added to the culture at 1 ng/ml final concentration. Lactococcal phages were propagated on their host strain grown to an optical density at 600 nm (OD_600__nm_) of 0.1 to 0.2 in GM17 broth supplemented with 10 mM CaCl_2_, as previously described (37).

### Membrane preparation from lactococci

*L. cremoris* strains were grown to an OD_600nm_ value of 1. Cells were harvested, washed in 25 mM 2-(N-morpholino)-ethane sulfonic acid (MES) pH 6.0, resuspended in the same buffer containing 5 mg mL^−1^ lysozyme (Sigma-Aldrich) and 10 units.mL^−1^ mutanolysin (Sigma-Aldrich), and incubated at 37 °C for 1 h. To isolate lactococcal membranes, cells were disrupted with one passage at 1.8 kbar in a One-Shot cell-disruptor system (CellD). Unbroken cells were removed by centrifugation at 5000*g* for 15 min at 4 °C and supernatants were then centrifuged at 150,000*g* for 60 min at 4 °C. The pellets containing membranes were resuspended in 50 mM Tris-HCl, pH 7.3 supplemented with 0.25 M sucrose.

### DNA techniques

Genomic DNA was extracted from lactococci with Wizard Genomic DNA Purification Kit (Promega). Before extraction, bacteria were incubated in 10 mM Tris-HCl, pH 8.0 supplemented with 25% sucrose and 30 mg mL^−1^ lysozyme at 37 °C for 1 h. Plasmid DNA was extracted from *E. coli* with QIAgen Spin Miniprep Kit, and from *L. cremoris* with the same kit, with a 1 h-pre-incubation with lysozyme as described above. PCR products were amplified with Phusion High-Fidelity DNA Polymerase (NEB) in a Mastercycler (Eppendorf), digested with restriction enzymes (NEB) and purified with a NucleoSpin Gel and PCR CleanUp kit (Macherey-Nagel). Ligations were performed with Lucigen Fast-Link DNA Ligation Kit. Alternatively, fragments were assembled by the strand overlap extension method using Gibson Assembly Master Mix (NEB). All plasmid constructs were verified by sequencing (Eurofins) using the primers pairs, pET21-f/pET21-r, msp3545-f/msp3545-r and pMalE/pMal-rev for pET21, pNZ8048 and pMal-c4X vectors, respectively (Table S2).

### Plasmid construction for protein expression in *E. coli*

WpsA was produced in *E. coli* as an N-terminal His_6_-tagged protein from the pET21His30 expression vector. WpsA with an N-terminal His_6_-tag was also co-produced with WpsB with the same expression vector. Gene *wpsA* (*llnz_1135*) was amplified as a PCR product using the primer pair 218_His30-f and 218_His30-r (Table S2), and genes *wpsA* and *wpsB* (*llnz_1140*) were amplified by PCR as a single bicistronic DNA fragment using the primer pair 218_His30-f and 219_His30-r (Table S2), with NZ9000 genomic DNA as a template. Each of the resulting amplicons was digested with restriction enzymes BamHI and XhoI and ligated into vector pET21His30, linearized with the same enzymes. The resulting plasmids p-*wpsA* and p*-wpsAB* were introduced in *E. coli* C43(DE3) by electroporation.

A fusion protein (WpsBA^fu^) comprising WpsB fused to WpsA was produced in *E. coli* with an N-terminal His_6_-tag from pET21His30 expression vector. Genes *wpsB* and *wpsA* were amplified by PCR with the primer pairs fuBA-H30B-f and fuBA-B-r, and fuBA-A-f and fuBA-H30A-r (Table S2), respectively, and with NZ9000 genomic DNA as a template. The two amplicons were assembled with pET21His30 vector linearized with restriction enzymes BamHI and XhoI using Gibson Assembly Master Mix (NEB). The resulting plasmid p-*wpsBAfu* was introduced in *E. coli* C43(DE3) by electroporation.

NZ_WpsC was produced in *E. coli* as a C-terminal His_6_-tagged protein from pET21His60 expression vector. Gene *wpsC* (*llnz_1145*) was PCR amplified using the primer pair NZ_wpsC-f and NZ_wpsC-r (Table S2) and with *L. cremoris* NZ9000 genomic DNA as a template. The amplicon was digested with NcoI and BglII and ligated into plasmid vector pET21His60, linearized with the same enzymes. The resulting plasmid p-*NZ_wpsC* was introduced into *E. coli* C43 (DE3) by electroporation.

SK_WpsC was produced in *E. coli* as a fusion protein with the maltose-binding protein (MBP) at its N-terminus from pMalC4X expression vector. Gene *wpsC* (*LACR_0215*) was amplified by PCR using the primer pair Sk_wpsC-f and Sk_wpsC-r (Table S2) with *L. cremoris* SK11 genomic DNA as a template. The amplicon was digested with restriction enzyme EcoRI and ligated into plasmid vector pMalC4X, linearized with XmnI and EcoRI. The resulting plasmid p-*SK_wpsC* was introduced in *E. coli* BL21 by electroporation.

### Expression and purification of recombinant proteins

*E. coli* C43(DE3) containing p-*wpsA*, p-*wpsAB*, p-*wpsBAfu*, p-*NZ_wpsC* or pGacIJ was grown to an OD_600nm_ of 0.6 to 0.8 in LB medium. *E. coli* BL21 containing p-*SK_wpsC* was grown to an OD_600nm_ of 0.6 to 0.8 in LB Amp supplemented with 0.2% glucose. Protein expression was induced by addition of 1 mM IPTG and further incubation of the cultures overnight at 18 °C. Cells were harvested, washed and resuspended in 25 mM Tris-HCl, 300 mM NaCl pH 7.3 buffer. Cells were then disrupted with a One-Shot cell disruptor (CellD), with one passage at 1.6 kbar. Unbroken cells were removed by centrifugation at 5000*g* for 15 min at 4 °C and the supernatant was recovered. This supernatant was further centrifuged at 20,000*g* for 20 min at 4 °C to obtain the soluble protein fraction. Alternatively, it was centrifuged at 150,000*g* for 60 min at 4 °C and the pellet containing membranes was recovered.

His_6_-tagged NZ_WpsC was purified from the soluble protein fraction on a 1-mL Ni-NTA column (Cytiva) equilibrated with 25 mM Tris-HCl, 500 mM NaCl, 20 mM imidazole, pH 7.5 and connected to an ÄKTA go chromatography system (Cytiva) at 4 °C. Proteins were eluted with a 30-min gradient to reach 500 mM imidazole concentration with detection at 280 nm. Protein-containing fractions were analyzed on a 12% SDS-PAGE. Fractions containing NZ_WpsC were pooled and desalted on a PD-10 column (Cytiva) with 25 mM Tris-HCl, 150 mM NaCl, pH 7.5 supplemented with 10% glycerol as an eluant.

MBP-SK_WpsC fusion protein was purified from the soluble protein fraction on a 1-mL MBP-trap column (Cytiva) equilibrated with 25 mM Tris-HCl, 300 mM NaCl, pH 7.5 and connected to an ÄKTA go chromatography system (Cytiva) at 4 °C. MBP-fusion proteins were eluted with 10 mM maltose with detection at 280 nm. Fractions were collected and analyzed on 12% SDS-PAGE. Fractions containing MBP-SK_WpsC were pooled and concentrated with a centrifugal concentrator (Amicon Ultra Centrifugal Filter unit; cut-off 30 kDa) at 4 °C. Buffer was exchanged against 25 mM Tris 150 mM NaCl pH 7.5 supplemented with 10% glycerol.

The membrane fractions containing WpsA, WpsA and WpsB, GacI and GacJ or WpsBA^fu^, obtained after centrifugation at 150,000*g* as described above, were resuspended in 50 mM Tris-HCl, pH 7.5 supplemented with 0.25 M sucrose.

To solubilize His_6_-tagged WpsBA^fu^ fusion protein from the membrane fraction, the pellet obtained after ultracentrifugation was incubated with 2% CHAPS (Euromedex) overnight at 4 °C with gentle stirring. Insoluble material was removed by ultracentrifugation at 150,000*g* for 60 min at 4 °C. Solubilized His_6_-tagged WpsBA^fu^ was purified on a 1-mL Ni-NTA column and subsequently desalted as described above for NZ_WpsC with the addition of 1% CHAPS in all buffers.

Proteins in membrane fractions were quantified with Pierce BCA Protein assay kit (Thermo) following the manufacturer’s instructions. Purified proteins were quantified with a Nanodrop 2000 apparatus (Thermoscientific) by measuring absorbance at 280 nm, using a theoretical ε value calculated by the ProtParam software (https://web.expasy.org/protparam/).

The purification yields of the different recombinant proteins, their exact amino acid sequences and their respective calculated molecular extinction coefficients are presented in Fig. S12.

### SDS-PAGE and Western Blot

Proteins were loaded on 12% polyacrylamide gel, with color Prestained Protein Standard (NEB). After migration, proteins were stained with Instant Blue (Abcam). For Western blotting, proteins were transferred onto a nitrocellulose membrane (Amersham Protran, Cytiva) using a Power Blotter apparatus (Invitrogen). Membranes were then saturated in PBS containing 0.1% Tween 20 (PBST) and 4 % skim milk overnight. They were incubated for 2 h with PBST containing a 1:2000 dilution of mouse monoclonal anti-polyHistidine antibody (Sigma-Aldrich) or alternatively with mouse monoclonal anti-MBP antibody (NEB), and then with PBST containing a 1:1000 dilution of Goat anti-mouse IgG Peroxydase conjugated (Invitrogen) for 1 h. Immunodetected proteins were revealed with Pierce ECL Western Blotting Substrate (Thermo), following the manufacturer’s instructions, and chemiluminescence was detected with a ChemiDoc XRS+ system (Bio-Rad).

### dTDP-rhamnose synthesis

Synthesis of dTDP-L-rhamnose was performed as previously described (32) and was a kind gift of Pr. Chris Whitfield (University of Guelph, Canada). Briefly, a 5-mL reaction mixture containing 4 mM dTDP-D-glucopyranose, 0.2 mM NAD^+^ and 20 mM ammonium formate in 0.1 M Tris-HCl, pH 7.0 was incubated for 2 h at 37 °C with 250 μg/ml each of purified RmlB, RmlC and RmlD (Graninger *et al*., 1999), and 3.5 units of formate dehydrogenase from *Candida boidinii* (Sigma). Following incubation, proteins were removed by ultrafiltration in a 3000 MWCO Vivaspin filtration unit (Sartorius Biolab Products). Synthesis of the final reaction product, dTDP-L-Rha*p*, was confirmed by mass spectrometry using an Agilent LC-UHD Q-TOF instrument, operated in negative mode, in the Mass Spectrometry Facility at the University of Guelph Advanced Analysis Centre. The mass spectrum revealed a single major peak at *m/z* 547.07, which is the expected mass for dTDP-L-Rha*p*. Since the reaction resulted in essentially quantitative conversion of substrate to product, no further purification was performed and the protein-free reaction mixture was used directly in GT assays.

### Glycosyltransferase *in vitro* activity assays

To assess the activity of WpsA produced in *L. cremoris* or *E. coli* membranes and of purified recombinant WpsBA^fu^, membrane fractions (containing 30–100 μg of proteins) or pure recombinant protein (1.2 μg), were mixed with 250 μM of C_55_P (obtained from the Institute of Biochemistry and Biophysics of the Polish Academy of Sciences), 20 μM radiolabeled UDP-[^14^C]GlcNAc (Isobio, 55 mCi/mmol), 180 μM UDP-GlcNAc (Sigma-Aldrich), 20 mM MgCl_2_ and 1% CHAPS in 50 mM Tris-HCl, pH 7.5, in a total volume of 30 μl.

To assess the activity of purified recombinant NZ_WpsC and MBP-SK_WpsC, radiolabeled [^14^C]GlcNAc-P-C_55_ and unlabeled GlcNAc-P-C_55_ (approximatively 20 nmoles of each) were synthesized as described above with *E. coli* membranes expressing WpsA and WpsB, to be used as acceptor substrates. They were extracted from the respective reaction mixtures by addition of 0.5 volume H_2_O, 0.5 volume 1 M pyridinium acetate pH 4.5 and two volumes of 1-butanol to the reaction mixture. The mixtures were vortexed for 3 min and centrifuged for 10 min at 16,000*g* and the resulting upper butanol phases were recovered. Purified NZ_WpsC (6 μg) or purified MBP-SK_WpsC (30 μg) were mixed with an estimated concentration of 40 μM of extracted [^14^C]GlcNAc-P-C_55_ lipid and with 200 μM dTDP-Rha or 200 μM UDP-Gal (Sigma-Aldrich), respectively, 10 mM MgCl_2_ and 0.6% DMSO in 50 mM Tris-HCl, pH 7.5 in a final volume of 50 μl. The activity of MBP-SK_WpsC was also assessed with UDP-[^14^C]Gal (PerkinElmer, 258 mCi/mmol) and unlabeled GlcNAc-P-C_55_ incubated in the same conditions.

Following overnight incubation at 37 °C, the reaction mixtures were analyzed by thin-layer chromatography (TLC) on 60 F254 silica gel coated plates (Merck) using H_2_O:n-propanol:ammonium hydroxide (60:30:10, v/v/v) as the mobile phase. After migration, radioactive spots were detected after exposure of the silica plate on a storage phosphor screen and scanning with a Typhoon PhosphorImager (Cytiva/GE Healthcare).

In parallel, the same enzymatic reactions were performed with non-radiolabeled donor and acceptor substrates and lipids were extracted by the addition of 0.5 volume of H_2_O, 0.5 volume of 1 M pyridinium acetate pH 4.5 and two volumes of 1-butanol to the reaction mixture as described above. The resulting upper butanol phase was recovered and analyzed by LC-MS.

### LC-MS analysis of C_55_P and derivatives

C_55_P and derivatives were analyzed by LC-MS using an UHPLC instrument (Vanquish Flex, Thermo Scientific) connected to a Q Exactive Focus mass spectrometer (Thermo Fisher Scientific) (facilities located at ChemSyBio, Micalis, INRAE, Jouy-en-Josas). Samples were diluted 10-fold in buffer B with 2 μl injected onto a HILIC column (ACQUITY UPLC BEH Amide column; 100 by 2.1 mm; particle size 1.7 μm; Waters). Buffer B contained acetonitrile and 20 mM ammonium acetate, pH 3.2 (80:20, V/V) and buffer A contained 20 mM ammonium acetate pH 3.2. Elution was performed at a flow rate of 0.3 ml min^−1^ using an isocratic step of 100% buffer B for 2 min followed by a gradient to 90% of buffer B in 6 min then a washing step for 2 min at 10% buffer B followed by a re-equilibration step to the initial condition for 10 min for the next injection. Mass analysis was performed in HESI negative mode with an acquisition range of 700 to 1600 *m/z*. The extracted ion chromatograms (EICs) were realized using the Qual Browser suite (Thermo Xcalibur ver. 4.1.31.9, June 2017) with an accurate extraction window for each mass of compounds of interest (*m/z* corresponding to [M-H]^-^ +/− 20 ppm).

### Selection of NZ9000 *wpsC*

Strain NZ9000-GT1 (NZ9000 with an in-frame stop codon inserted in *wpsC (llnz_01145)* gene) obtained by recombineering as described previously (12) is resistant to chloramphenicol because of the presence of plasmid pJP005 (38). This strain was cultured by eight successive re-inoculations in liquid GM17 medium, then streaked out to single colonies on GM17 agar plates. A chloramphenicol-sensitive clone was selected and named NZ9000 *wpsC* (VES7971). The presence of the *wpsC* mutation was verified by PCR amplification and DNA nucleotide sequence determination.

### Complementation of *L. cremoris* NZ9000 *wpsC* and *wpsE* mutants

Genes *wpsC* (*smq17*) and *wpsD* (*smq18*) from *L. cremoris* SMQ-388 were amplified by PCR as a single bicistronic DNA fragment using the primer pair SMQ17-f and SMQ18-r (Table S2). The amplicon was assembled with plasmid pNZ8048, linearized with *Nco*I and *Pst*I, using Gibson Assembly Master Mix (NEB). The resulting plasmid p-*CD*_*SMQ*_ contained *wpsC* and *wpsD* genes from strain SMQ-388 under the control of a nisin inducible promoter. The plasmid was introduced in NZ9000 *wpsC* mutant by electroporation to obtain strain NZ *wpsC* (p-*CD*_*SMQ*_). Plasmid p-*CD*_*NZ*_ for complementation of NZ9000 *wpsC* mutant with *wpsC* (*llnz_1145*) and *wpsD* (*llnz_1150*) genes from *L. cremoris* NZ9000 was constructed following the same procedure using the primer pair NZ1145-f and NZ1150-r (Table S2). The plasmid was introduced in NZ9000 *wpsC* mutant by electroporation to obtain strain NZ *wpsC* (p-*CD*_*NZ*_).

Genes *wpsE* (*smq19*) and *wpsF* (*smq20*) from *L. cremoris* SMQ-388 were amplified by PCR as a single bicistronic DNA fragment using the primer pair SMQ19-f and SMQ20-r (Table S2). The amplicon was assembled with plasmid pNZ8048, linearized with *Nco*I and *Pst*I, using Gibson Assembly Master Mix (NEB). The resulting plasmid p-*EF_SMQ_* contained *wpsE* and *wpsF* genes from strain SMQ-388 under the control of a nisin inducible promoter. The plasmid was introduced in NZ9000 *wpsE* mutant by electroporation to obtain strain NZ *wpsE* (p-*EF*_*SMQ*_). Plasmid p-*EF*_*NZ*_ for complementation of NZ9000 *wpsE* mutant with *wpsE* (*llnz_1155*) and *wpsF* (*llnz_1160*) genes from *L. cremoris* NZ9000 was constructed following the same procedure using the primer pair NZ1155-f and NZ1160-r (Table S2). The plasmid was introduced in NZ9000 *wpsC* mutant by electroporation to obtain strain NZ *wpsE* (p-*EF*_*NZ*_).

### Phage sensitivity assay

Phage suspensions were diluted in sodium-magnesium (SM) buffer containing CaCl_2_ (100 mM NaCl, 10 mM MgSO_4_, 20 mM CaCl_2_, 10 mM Tris-HCl, pH 7.0) and added to overnight bacterial cultures diluted in semi-solid GM17 containing 0.4% agar, before plating on top of GM17 plates containing 1% agar as previously described (39). Semi-solid and solid GM17 were supplemented with 10 mM CaCl_2_. Lysis plaques were counted after an overnight incubation at 30 °C. The efficiency of plating (EOP) was determined as the ratio of the titer obtained on the tested strain to that obtained on the WT strain.

### TEM

Bacterial cells were fixed and embedded in Epon (DELTA Microscopies, France) as described previously (33). Thin sections (70 nm) were collected onto 200-mesh copper grids and counterstained with lead citrate. Grids were examined with a Hitachi HT7700 electron microscope operated at 80 kV, and images were acquired with a charge-coupled device camera (Advanced Microcopy Techniques) (facilities located on the MIMA2 platform, INRAE, Jouy-en-Josas, France; https://doi.org/10.15454/1.5572348210007727E12).

### CWPS extraction, purification and analysis

Bacteria were harvested from an exponentially growing culture at an OD_600nm_ of 0.6 and cell walls were prepared as described previously (33). CWPS (including rhamnan and PSP) were extracted by treatment with 48% HF for 48 h at 4 °C as described previously (10). Rhamnan and PSP oligosaccharides were separated by SEC–HPLC with two columns in tandem (Shodex Sugar KS-804 and KS-803 columns, 300 × 8 mm) as described previously (10). Elution was performed with Milli-Q H_2_O, and detection of eluted compounds was performed with a refractometer (2414 Refractive Index Detector, Waters) and a UV detector at 206 nm. Fractions containing PSP oligosaccharides were collected and dried under vacuum. They were further analyzed by Matrix Assisted Laser Desorption Ionization - Time of Flight (MALDI-TOF) MS using 2,5-dihydroxybenzoic acid (DHB) matrix with an UltrafleXtreme instrument (Bruker Daltonics) (facilities located at CEA, Médicaments et Technologies pour la Santé (MTS), MetaboHUB, Gif-sur-Yvette, France).

### WpsA/WpsB complex modeling

A Github/Colab AlphaFold2 v3.2.1 notebook was used to perform the predictions (https://colab.research.google.com/github/deepmind/alphafold/blob/main/notebooks/AlphaFold.ipynb#scrollTo=XUo6foMQxwS2). Local Distance Difference Test (LDDT) evaluates local distance differences of all atoms in a model with reference to an ensemble of equivalent structures. The pLDDT (predicted lDDT-Cα) is a per-residue measure of local confidence on a scale from 0 to 100 (100 being the highest confidence level). The pLDDT values that are stored in the pdb file as B-factors, were plotted using Excel (Fig. S2). The final predicted domain structures were submitted to the Dali server (28) to identify the closest structural homologs in the PDB. Coot (40) option “SSM Superpose” was used to superimpose WpsA/B structure onto the Dali hits. The ligands were extracted from PDB 5MM1 (29). Sequence alignments were performed with Multalin (41) and ESPript (42). Visual representations of the structures were prepared with ChimeraX (27). Analyses of protein-protein interfaces were performed using the PDBePISA server (25).

## Data availability

All coordinates of predicted structures are available as supporting information.

## Supporting information

This article contains supporting information (12, 14, 15, 16, 23, 36, 43, 44, 45, 46, 47, 48, 49, 50, 51).

## Conflicts of interest

C. C. is an employee of Alphagraphix (cambillau.alphagraphix@gmail.com). Authors and Alphagraphix declare that they have no competing interests. The funders had no role in the design of the study; in the collection, analyses, or interpretation of data; in the writing of the manuscript; or in the decision to publish the results.
