# Supplementary material for: Molecular mechanisms underlying the structural diversity of rhamnose-rich cell wall polysaccharides in lactococci
Source: J Biol Chem. 2023 Dec 16;300(1):105578. doi: 10.1016/j.jbc.2023.105578 (PMC10821137; doi:10.1016/j.jbc.2023.105578)
Supplement: Supporting information [file mmc1.docx]

**Supporting information**

**Molecular mechanisms underlying the structural diversity of rhamnose-rich cell wall polysaccharides in lactococci**

Hugo Guérin^1^, Pascal Courtin^1^, Alain Guillot^1^, Christine Péchoux^2^, Jennifer Mahony^3^, Douwe van Sinderen^3^, Saulius Kulakauskas^1^, Christian Cambillau^3,4^, Thierry Touzé^5^ and Marie-Pierre Chapot-Chartier^1^

^1^Université Paris-Saclay, INRAE, AgroParisTech, Micalis Institute, Jouy-en-Josas, France

^2^Université Paris-Saclay INRAE, AgroParisTech, GABI, Jouy-en-Josas, France

^3^School of Microbiology and APC Microbiome Ireland, University College Cork, Western Road, Cork, Ireland

^4^Laboratoire d’Ingénierie des Systèmes Macromoléculaires (LISM), Institut de Microbiologie, Bioénergies et Biotechnologie (IMM), Aix-Marseille Université – CNRS, UMR 7255, Marseille, France.

^5^Université Paris-Saclay, CEA, CNRS, Institute for Integrative Biology of the Cell (I2BC), Gif-sur-Yvette, France.

**List of supplementary material**

**Figure S1**. SDS-PAGE and Western Blot analysis of the recombinant proteins used in this study.

**Figure S2.** AlphaFold2 pLDDT values and predicted aligned error of the WpsA/WpsB complex model

**Figure S3.** PISA analysis of WpsA/WpsB interactions.

**Figure S4.** TMHMM transmembrane helix prediction for WpsA and WpsB.

**Figure S5.** Dali search with WpsA amino-acid sequence as a query.

**Figure S6.** WpsBA^fu^ fusion protein activity assay.

**Figure S7.** SEC-HPLC analyses of CWPS extracted from *L. cremoris* NZ9000 and derivative mutants.

**Figure S8.** MALDI-TOF mass spectra of the purified PSP oligosaccharides from *L. cremoris* NZ9000 and mutants NZ9000 *wpsE*, NZ *wpsE (*p-*EF^NZ^)* and NZ *wpsE (*p-*EF^SMQ^).*

**Figure S9.** Primary sequences alignement of lactococcal WpsA and WpsB with S. *pyogenes* GacI and GacJ, and homologs present as a unique fusion protein in several species.

**Figure S10.** Sequence alignment of polyisoprenyl-phosphate glycosyltransferases.

**Figure S11.** Structure of the polymeric PSP of *L. cremoris* NZ9000, SMQ-388 and 3107.

**Figure S12.** Amino acid sequence, molar extinction coefficient (ε) and purification yield of each of the three recombinant proteins purified from *E. coli.*

**Table S1.** Strains, plasmids, and bacteriophages used in this study.

**Table S2.** Primers used in this study.


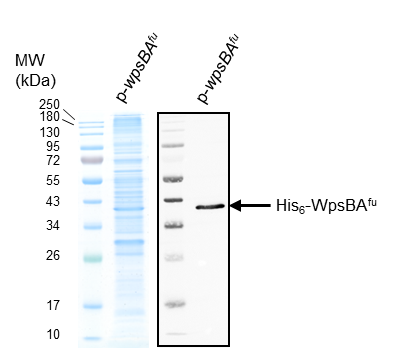

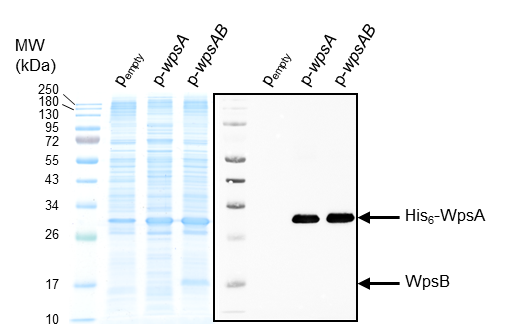


**A**

**C**


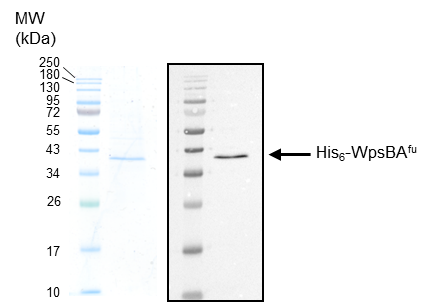


**B**

**D**


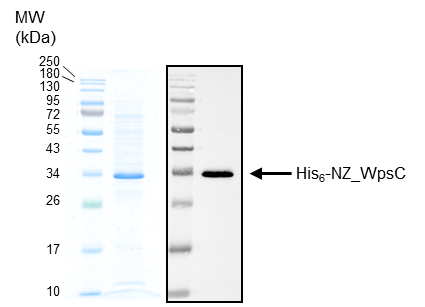

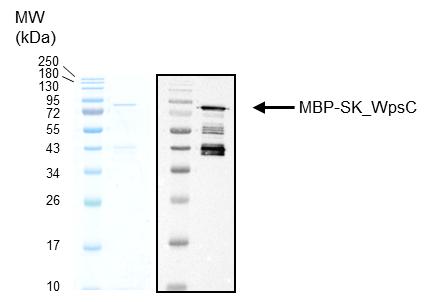


**E**

**Figure S1. SDS-PAGE and Western Blot analysis of the recombinant proteins used in this study**. (A) and (B), membrane proteins (10 µg) of IPTG-induced *E. coli* recombinant strains with the indicated plasmids; (C), (D) and (E): Purified recombinant proteins (2 µg). Western blots were probed with anti-polyHistidine antibody (A, B, C and D) or anti-MBP antibody (E). Expected molecular weight: His_6_-WpsA, 27.9 kDa; WpsB, 13.3 kDa; His_6_-WpsBA^fu^ , 40.7 kDa; His_6_-NZ_WpsC, 32.2 kDa; MBP-SK_WpsC, 88.7 kDa.


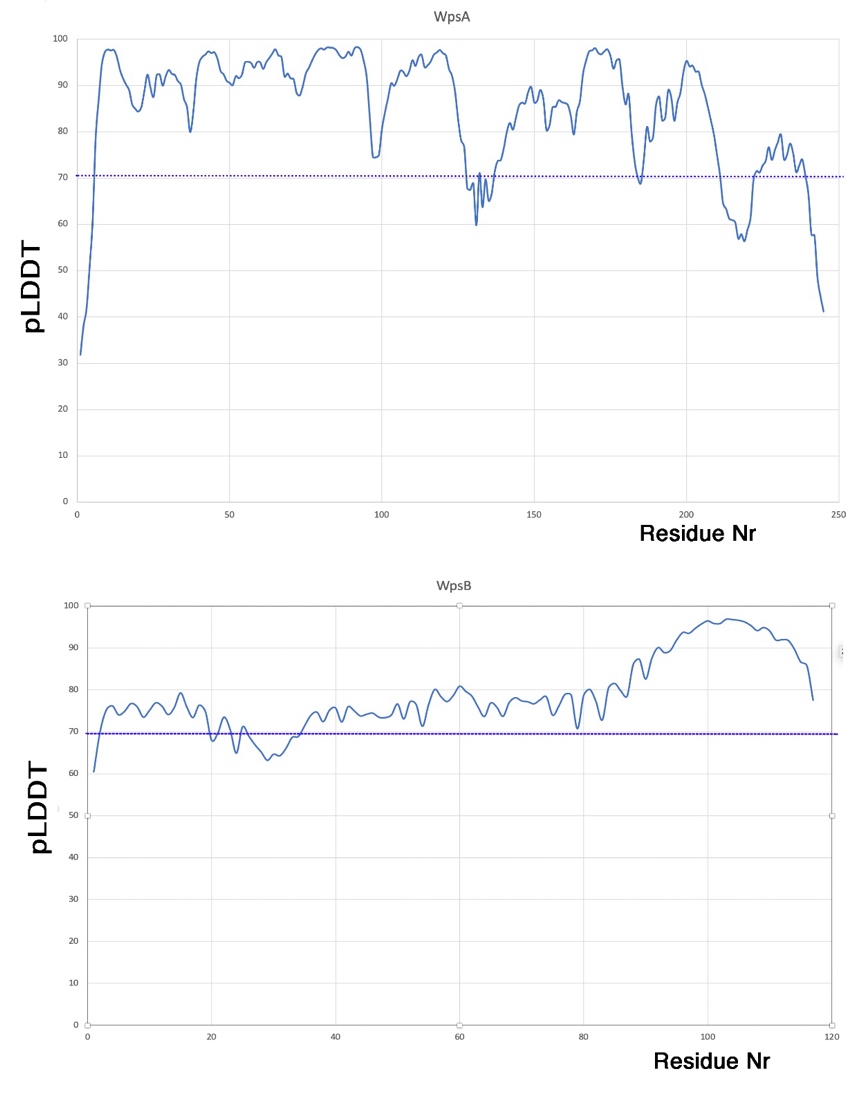


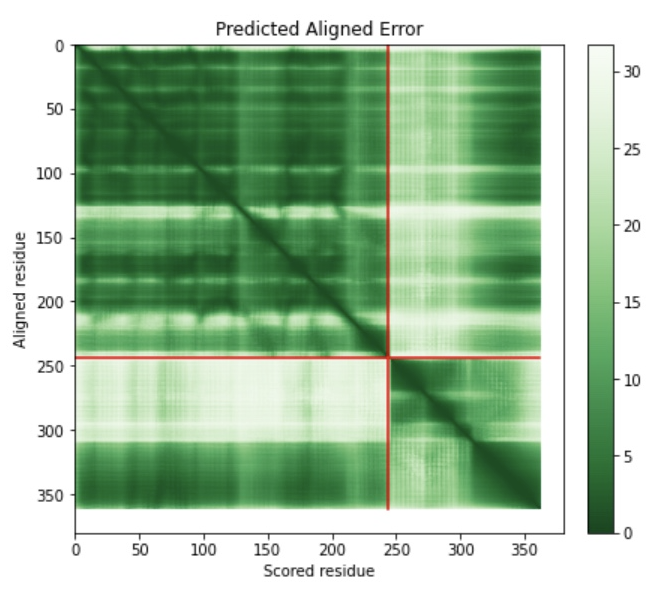


**Figure S2. Alphafold2 pLDDT values and predicted aligned error of the WpsA/WpsB complex model**


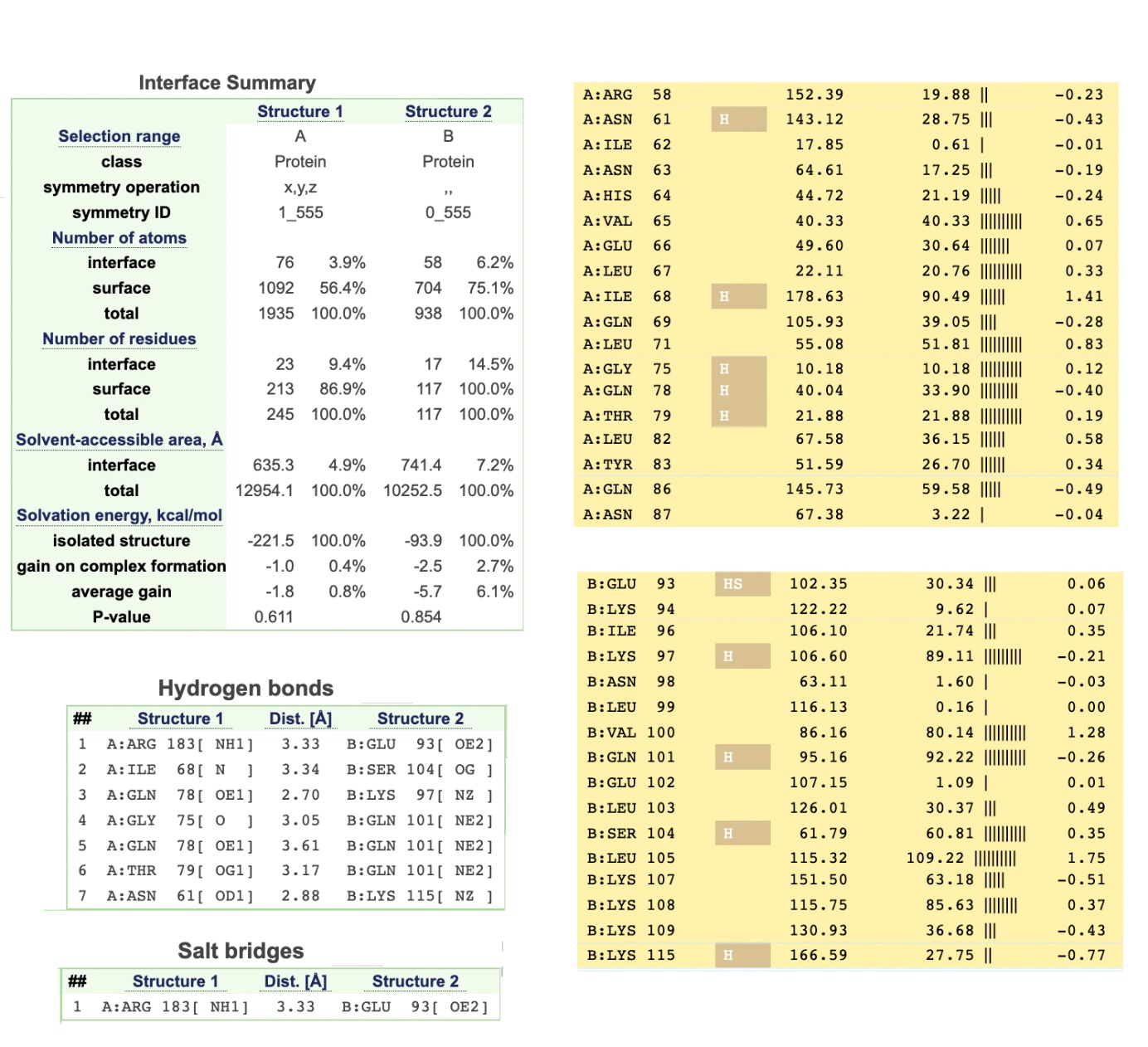


**Figure S3. PISA analysis of WpsA/WpsB interactions.**


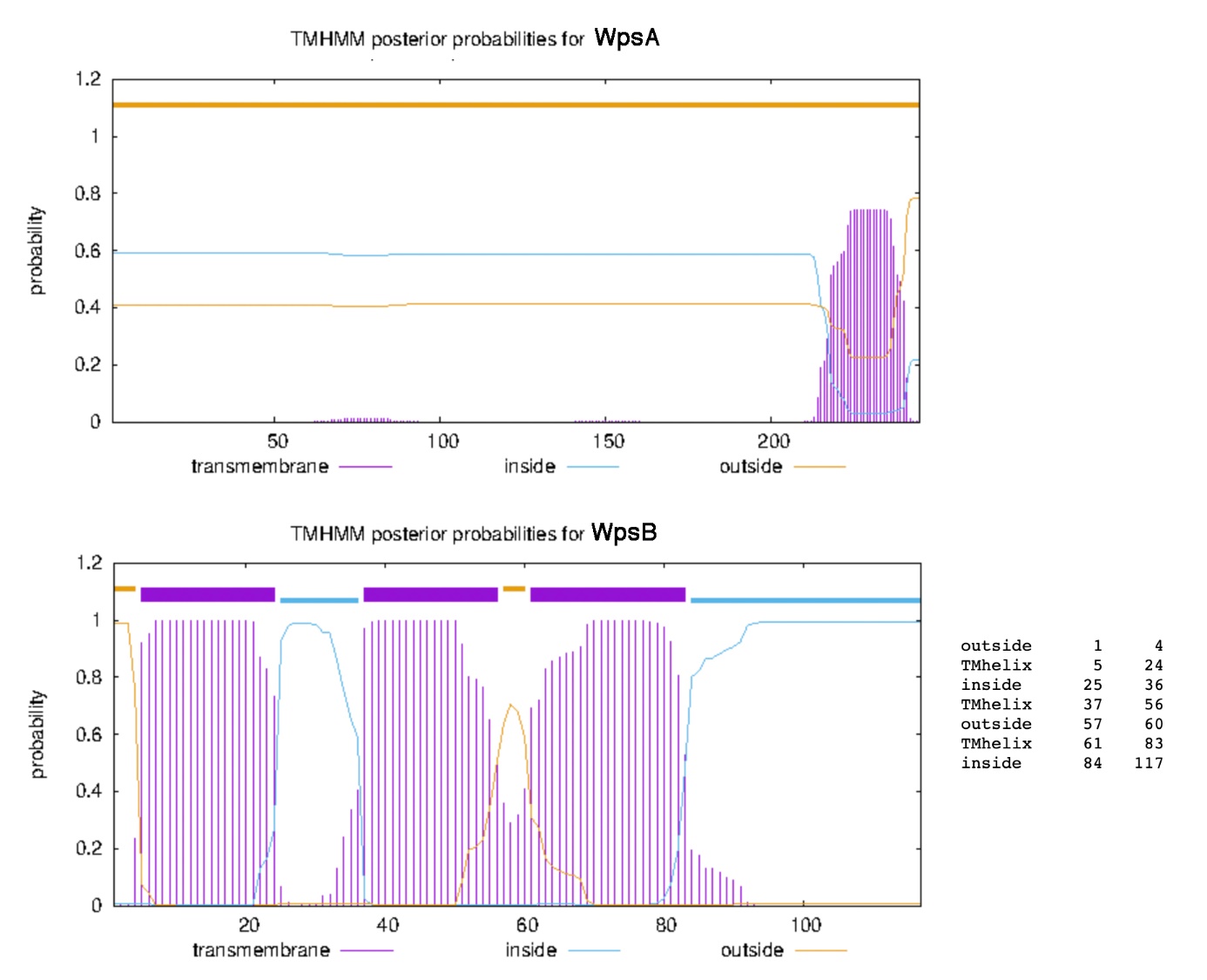


**Figure S4. TMHMM transmembrane helix prediction for WpsA and WpsB.**


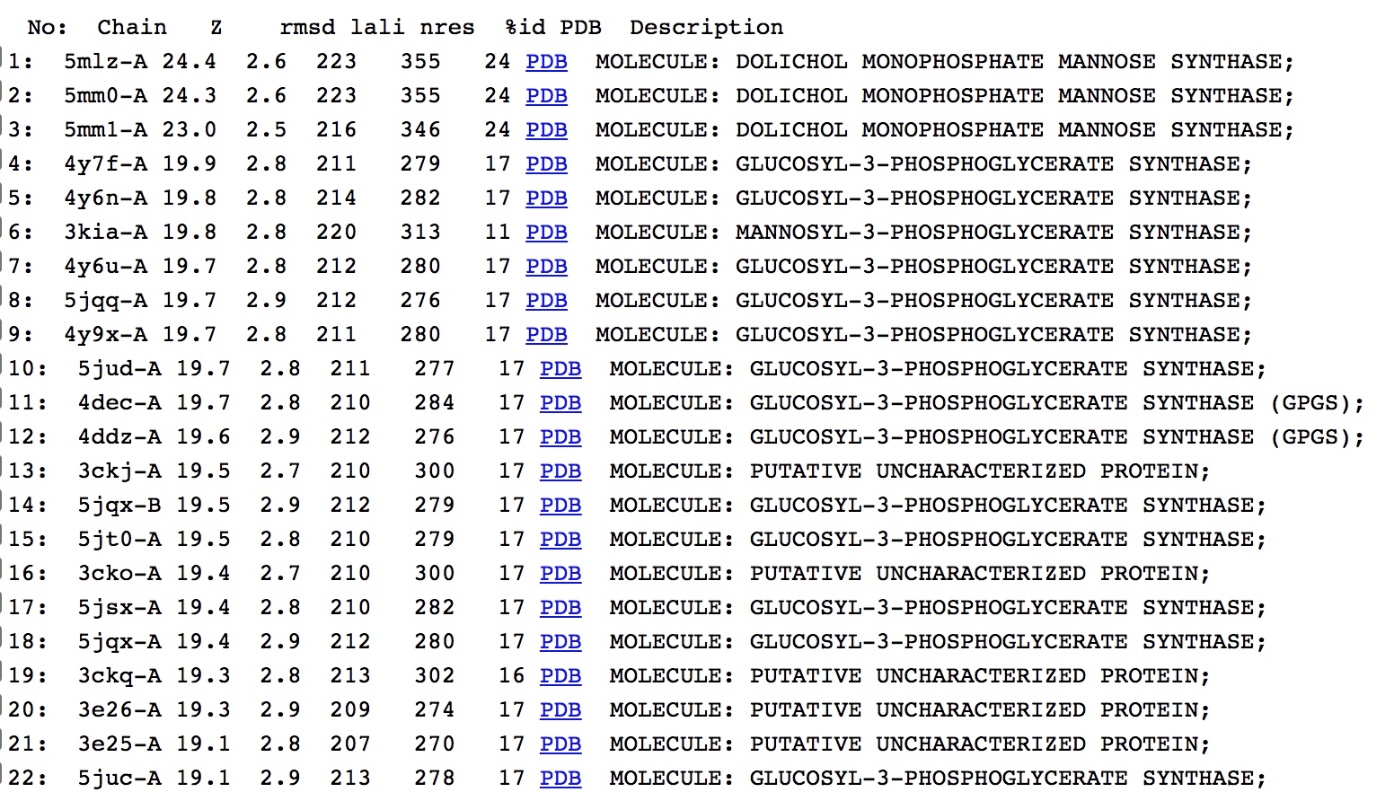


**Figure S5. Dali search with WpsA amino-acid sequence as a query.**


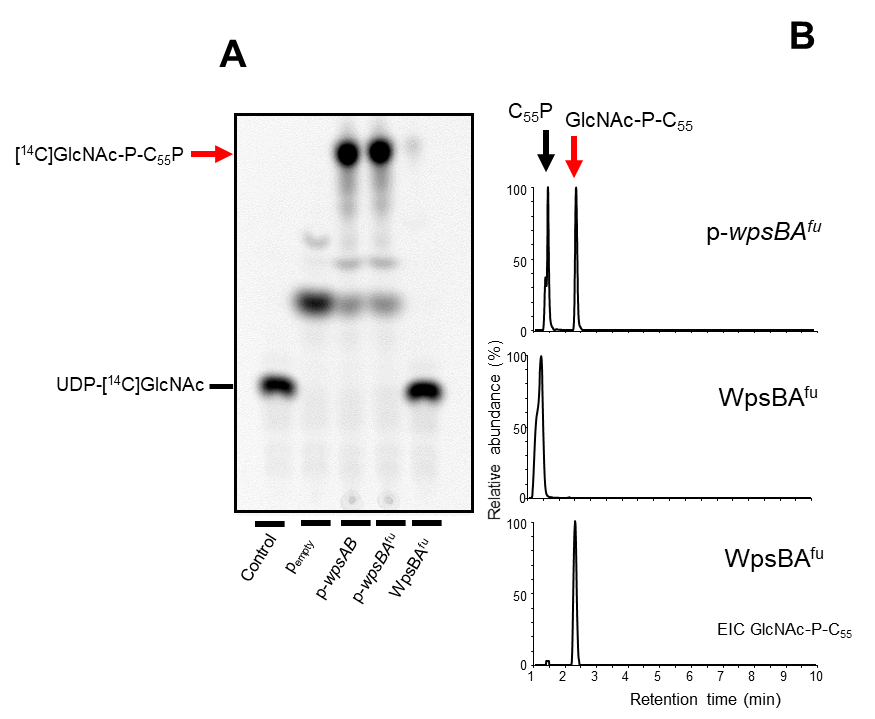


**Figure S6. WpsBA^fu^ fusion protein activity assay. (A)** TLC analysis of the reaction mixtures revealed by phosphorimager. Reaction mixtures contained membranes of *E. coli* with empty pET21His30 plasmid (p_empty_), p-*wpsAB* or p-*wpsBA^fu^* plasmid or purified WpsBA^fu^ fusion protein, UDP-[^14^C]GlcNAc donor and C_55_P acceptor substrates. Red arrow indicates the newly formed radiolabelled product identified as [^14^C]GlcNAc-P-C_55_ in *E. coli* membranes expressing *wpsA* and *wpsB*. **(B)** LC-MS analysis in the negative ion mode of butanol-extracted C_55_P derivatives. Combined EICs for C_55_P and GlcNAc-P-C_55_ ions ([M-H]^-^) ions are shown for *E. coli* containing p-*wpsBA^fu^* membranes (upper chromatogram) and pure WpsBA^fu^ (middle chromatogram) samples, and EIC of the sole GlcNAc-P-C_55_ ion for pure WpsBA^fu^ sample (lower chromatogram).

**Figure S7.** SEC-HPLC analyses of CWPS extracted from *L. cremoris* NZ9000 and derivative mutants. Peaks containing rhamnan and PSP oligosaccharides are indicated. * designate non-polysaccharidic compounds.


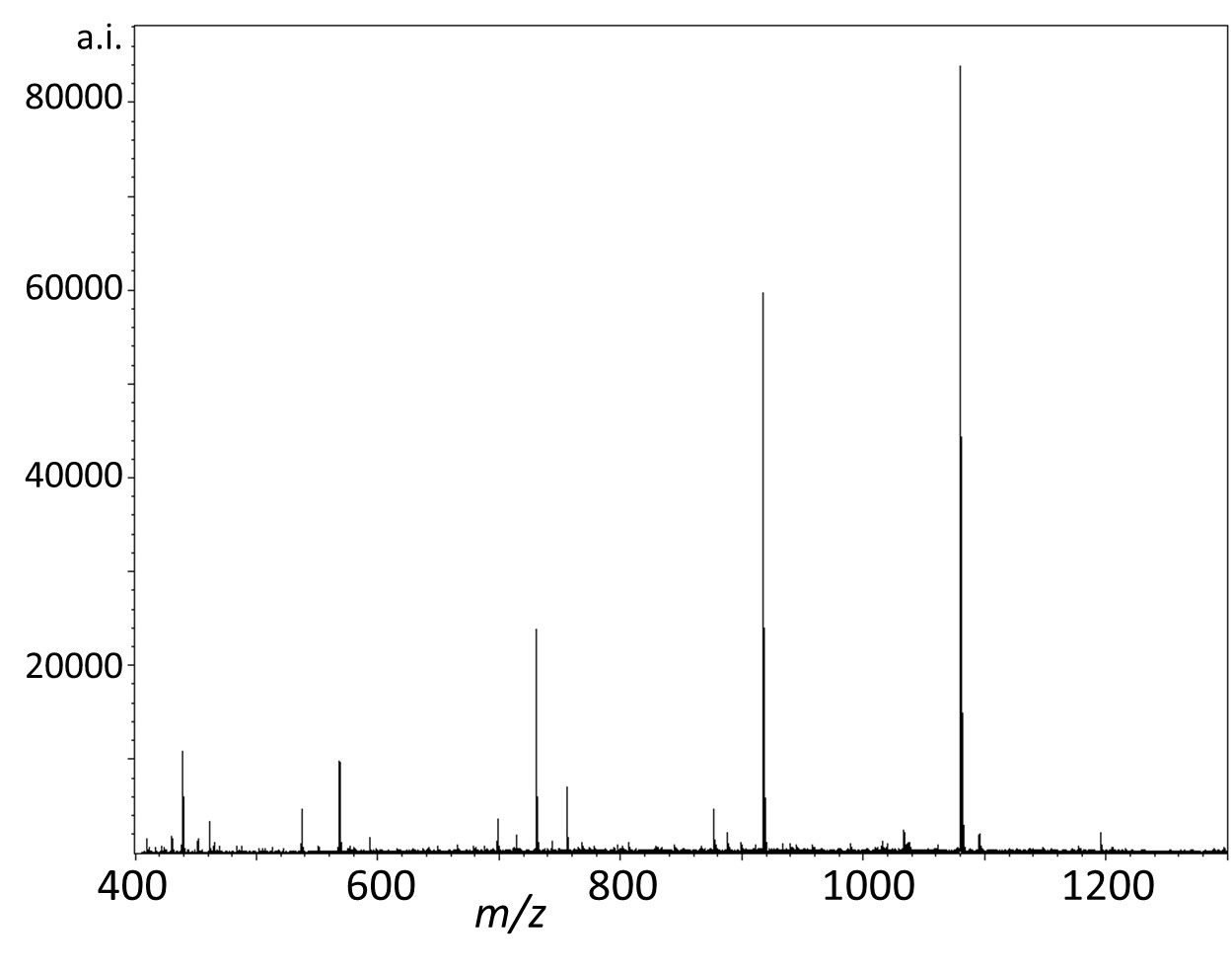


1079.07

917.05

**NZ9000**


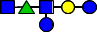


***f***


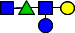


***f***


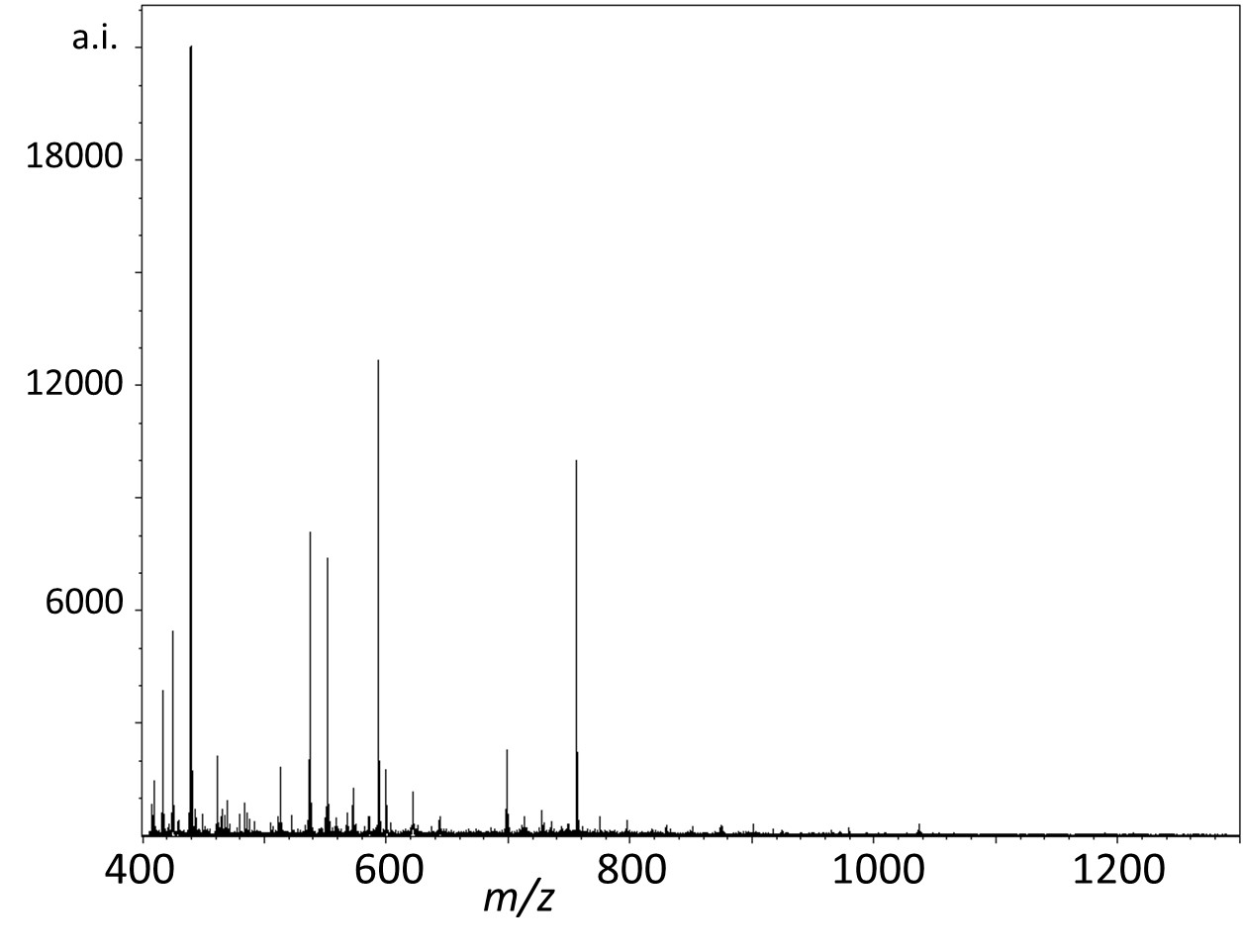


***wpsE***

755.03

593.06


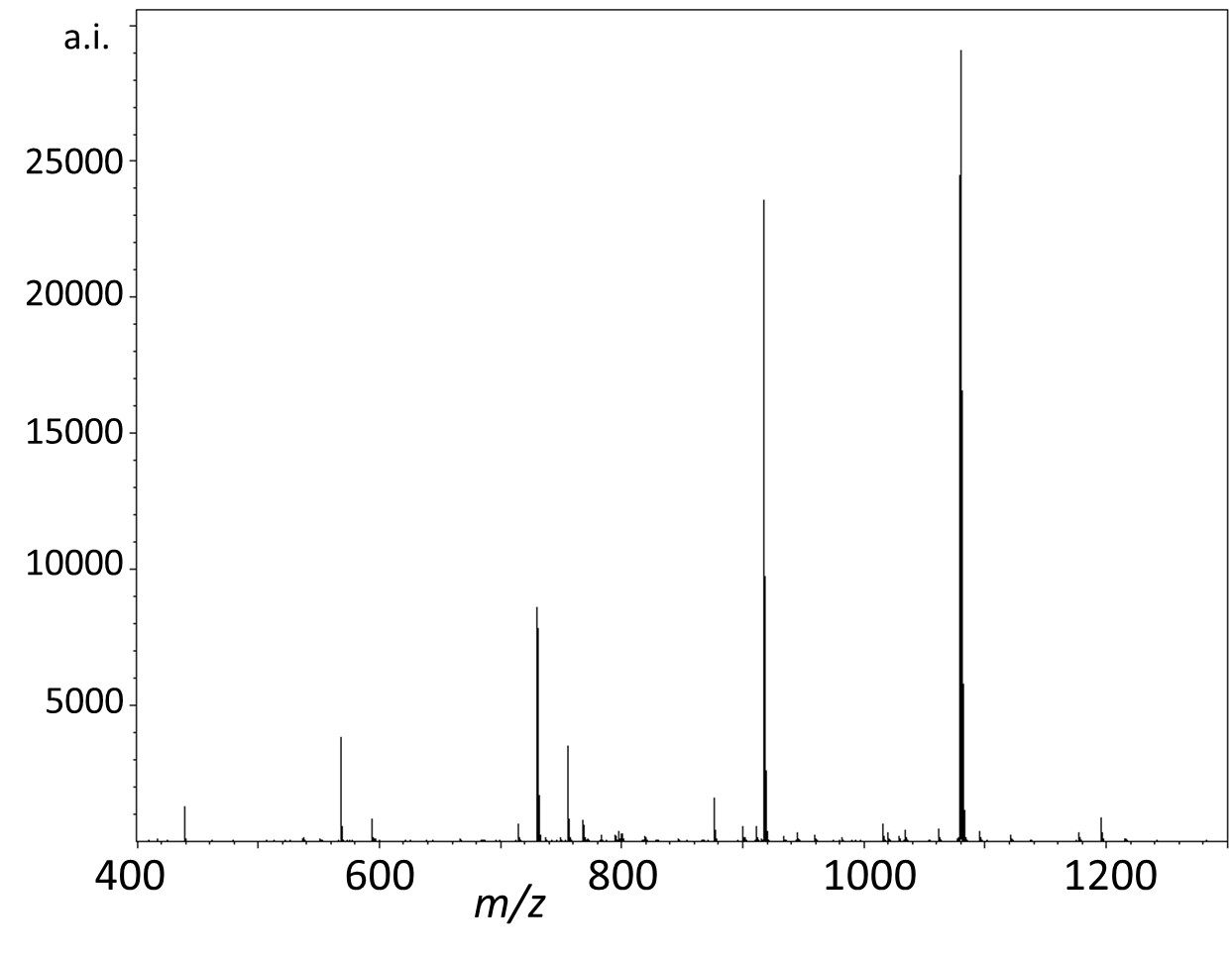


1079.16

917.12


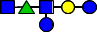


***f***


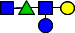


***f***

***wpsE* (p-*EF_NZ_*)**


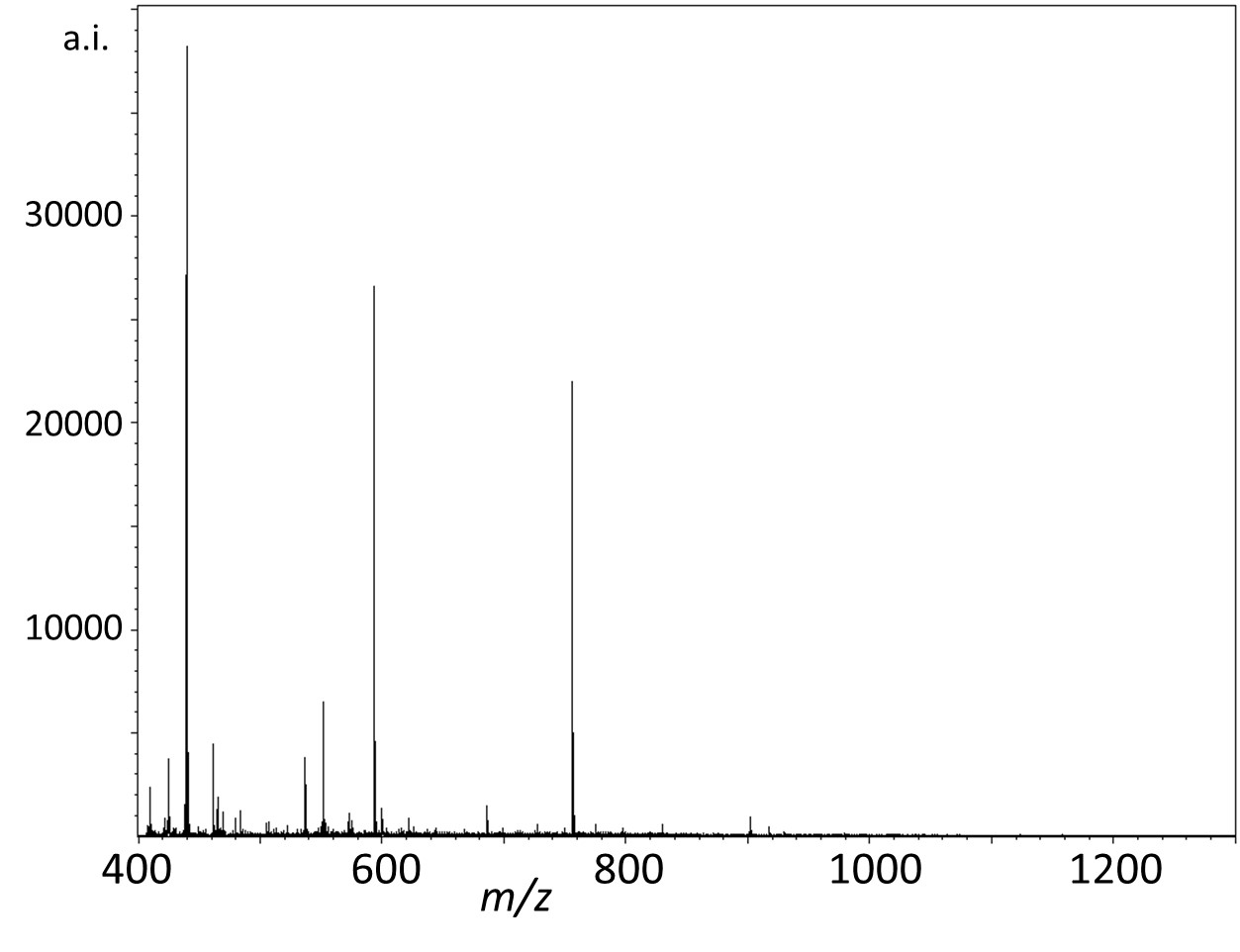


***wpsE* (p-*EF_SMQ_*)**

755.15

593.14

**Figure S8. MALDI-TOF mass spectra of the purified PSP oligosaccharides from *L. cremoris* NZ9000 and mutants.** *m/z* values correspond to [M+Na]^+^ adducts. *L. lactis* NZ9000 synthesizes polymeric PSP that is cleaved during HF extraction at the level of phosphodiester bonds leading to hexasacharide (calculated *m/z* [M+Na]^+^1079.37) and partially also between Gal*f* and Glc leading to a pentasaccharide (calculated *m/z* [M+Na]^+^ 917.32), as shown previously by NMR analysis (1). An identical spectrum is observed for NZ *wpsE* (p-*EF^NZ^*) PSP. Peaks detected in the NZ9000 *wpsE* spectrum have been assigned to remnant non-polymerized truncated PSP subunit fragments (2). No new peak is detected in mutant NZ *wpsE (*p-*EF_SMQ_)*. Blue square, GlcNAc; green triangle, Rha; blue circle, Glc; yellow circle, Gal; *f*, furanose. Note that the panel showing the WT NZ9000 spectrum is the same as the panel shown in Fig.7C. All the Maldi-Tof MS spectra shown on Fig. 7C and Fig. S8 were acquired in the same experimental series.


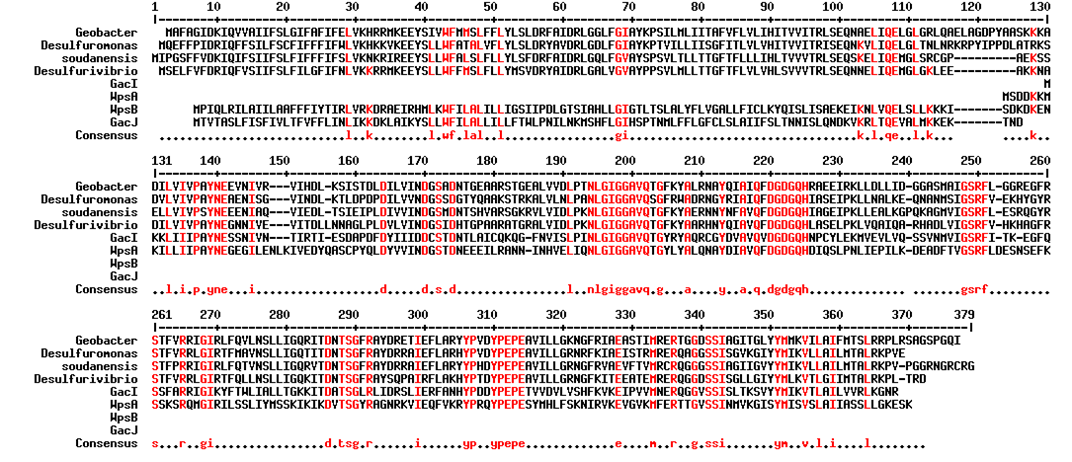


**Figure S9. Primary sequence alignement of lactococcal WpsA and WpsB with S. *pyogenes* GacI and GacJ, and homologs present as a unique fusion protein in several species.** Conserved aminoacids are in red.

DxDxQ

**Figure S10.** **Sequence alignment of polyisoprenyl-phosphate glycosyltransferases.**

*L. cremoris* WpsA, *S. pyogenes* GacI, *P. furiosus* DPMS, *L. cremoris* CsdE, CsdA and CsdC (3), *Listeria monocytogenes* GtlA (Lmo0933) (4) and Lm02550 (5), and *Synechocystis sp.* GtrB (6).

The conserved DxDxQ motif is boxed in green.

**
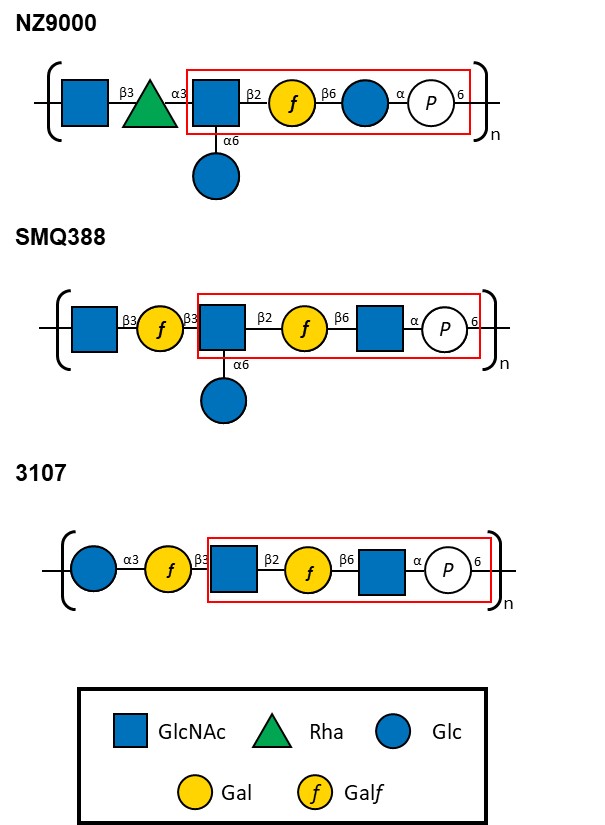
**

**Figure S11. Structure of the polymeric PSP of *L. cremoris* NZ9000, SMQ-388 and 3107.**

The three structures were established by NMR (1,7,8). The polymeric chains are represented in a way to make visible the semi-conserved trisaccharide motif shared by the three PSP repeats. The motif is boxed in red.

- **Fusion protein WpsBA**

Amino acid sequence of the recombinant protein

MHHHHHHGPIQLRILAIILAAFFFIYTIRLVRKDRAEIRHMLKWFILALILLIGSIIPDLGTSIAHLLGIGTLTSLALYFLVGALLFICLKYQISLISAEKEIKNLVQELSLLKKKISDKDKENKILLIIPAYNEGEGILENLKIVEDYQASCPYQLDYVVINDGSTDNEEEILRANNINHVELIQNLGIGGAVQTGYLYALQNAYDIAVQFDGDGQHDIQSLPNLIEPILKDEADFTVGSRFLDESNSEFKSSKSRQMGIRILSSLIYMSSKIKIKDVTSGYRAGNRKVIEQFVKRYPRQYPEPESYMHLFSKNIRVKEVGVKMFERTTGVSSINMVKGISYMISVSLAIIASSLLGKESK

ε (computed with ProtParam) : 29465 M^-1^ cm^-1^

Purification yield: 0.22 mg/L culture

- **NZ_WpsC**

Amino acid sequence of the recombinant protein

MGNKVIKHTFVICAYMQSPYLEESIKSILDQGSIKEGISEVVLYTSTPNDYIENICHKYNIKIFIGEGGGIGADWNGALAAVQTKYATIVHQDDLYDKKYGEMIINDFESQKDSNIVFTDYYEIDEYSKPRKRNINLKIKSLGLKLMSFWENKKYQRRVYSFGNFICCPAVSYNMERLKDFRFNEEMKMAVDWDAWERIMKKSGHVHYLPLKLMAHRIHSDSETTNNTLNKNREKEEHEMFRRYWGETMAKLLMKVYTNNQKGNSRSHHHHHH

ε (computed with ProtParam) : 53080 M^-1^ cm^-1^

Purification yield : 0.6 mg/L culture

- **SK_WpsC (MBP fusion)**

Amino acid sequence MKIKTGARILALSALTTMMFSASALAKIEEGKLVIWINGDKGYNGLAEVGKKFEKDTGIKVTVEHPDKLEEKFPQVAATGDGPDIIFWAHDRFGGYAQSGLLAEITPDKAFQDKLYPFTWDAVRYNGKLIAYPIAVEALSLIYNKDLLPNPPKTWEEIPALDKELKAKGKSALMFNLQEPYFTWPLIAADGGYAFKYENGKYDIKDVGVDNAGAKAGLTFLVDLIKNKHMNADTDYSIAEAAFNKGETAMTINGPWAWSNIDTSKVNYGVTVLPTFKGQPSKPFVGVLSAGINAASPNKELAKEFLENYLLTDEGLEAVNKDKPLGAVALKSYEEELAKDPRIAATMENAQKGEIMPNIPQMSAFWYAVRTAVINAASGRQTVDEALKDAQTNSSSNNNNNNNNNNLGIEGRKTVAIFSGFYLPFLGGIERYTYNIVQKFIDKGYNVVIVTSQHDKDLPTKEEFDHLKIYRLPIRKIWKNRYPFPLKNERYKQLISDITSEPIDYYVVNTRFQLPALLGAQLAKKAGKEALVLEHGTTYLTLNNSLLDSILHRIEHFLVKKIKKNTKTFYGVSKEATEWLKTFGIEAKGVIYNAIDLDDFEKYFSPKLSEKIVISYSGRLQAKFKGVEMLLEAFSELSKERNNLELKIAGDGPIYSEMIRKYSQENIQFLGHISHEDVMRLNNHSDIFVLMSKIEGFSTSMLEAALMNNVIVTTNVGGATELIPNQEYGYVIKDDKETLLKTLKLIVDDTKKMAEIQKRVHDRVVENFNWNKSIESFEKAFVELEKIEIN

ε (computed with ProtParam) : 109670 M^-1^ cm^-1^

Purification yield : 12 mg/L culture

**Figure S12.** **Amino acid sequence, molar extinction coefficient (ε) and purification yield of each of the three recombinant proteins purified from *E. coli.*** 6xHis-tag is indicated in green. Extra residues added for in frame cloning in the plasmid vector are indicated in red as well as the MBP sequence for the SK_WpsC protein. WpsB sequence is indicated in blue.

**Table S1.** **Strains, plasmids, and bacteriophages used in this study**

| **Bacterial strains, plasmids, or phages** | **Relevant genotype and/or characteristic(s)** | **Source or reference** |
| --- | --- | --- |
| ***L. cremoris*** |  |  |
| MG1363 | Plasmid-free strain | (9) |
| NZ9000 | MG1363 *pepN*::*nisRK* | (10) |
| NZ9000 *wpsA* | NZ9000 with two in-frame stop codons inserted in *wpsA (llnz_01135)* gene | (2) |
| NZ9000 *wpsB* | NZ9000 with two in-frame stop codons inserted in *wpsB (llnz_01140)* gene | (2) |
| NZ9000 *wpsC* | NZ9000 with an in-frame stop codon inserted in *wpsC (llnz_01145)* gene | (8) and this work |
| NZ9000 *wpsE* | NZ9000 with an in-frame stop codon inserted in *wpsE (llnz_01155)* gene | (2) |
| NZ *wpsC* (p-*CD_NZ_*) | NZ9000 *wpsC* mutant complemented with *wpsCD* genes from NZ9000 | This work |
| NZ *wpsC* (p-*CD_SMQ_*) | NZ9000 *wpsC* mutant complemented with *wpsCD* genes from SMQ388 | This work |
| NZ *wpsE* (p-*EF_NZ_*) | NZ9000 *wpsE* mutant complemented with *wpsEF* genes from NZ9000 | This work |
| NZ *wpsE* (p-*EF_SMQ_*) | NZ9000 *wpsE* mutant complemented with *wpsEF* genes from SMQ388 | This work |
| SMQ-388 | Wild-type strain | S. Moineau, Université Laval, Canada |
| SK11 | Wild-type strain | INRAE collection |
|  |  |  |
| ***E. coli*** |  |  |
| TG1 | *F' [traD36 proAB lacIqZ ΔM15] supE thi-1 Δ(lac-proAB) Δ(mcrB-hsdSM)5(rK - mK -)* |  |
| JIM4646 | TG1 strain with a chromosomal copy of *repA* gene, allowing replication of *L. lactis* pNZ8048 plasmid | P. Renault, INRAE, France |
| DH5alpha | *fhuA2 lac(del)U169 phoA glnV44 Φ80' lacZ(del)M15 gyrA96 recA1 relA1 endA1 thi-1 hsdR17* | Invitrogen |
| Top10 | *F- mcrA Δ(mrr-hsdRMS-mcrBC) φ80lacZΔM15 ΔlacX74 nupG recA1 araD139 Δ(ara-leu)7697 galE15 galK16 rpsL(Str^R^) endA1 λ^-^* | Invitrogen |
| C43(DE3) | F-*ompT, gal, hsdsB,*(rB-mB-) *dcm, DE3* | Avidis |
| BL21 | *B dcm ompT hsdS*(r_B_^-^m_B_^-^) *gal* | NEB |
|  |  |  |
| **Plasmids** |  |  |
| pET21His30 | *E. coli* expression pET21d derivative vector for N-terminal His_6_-tag fusion, T7 promoter, Amp^R^ | (11) |
| pET21His60 | *E. coli* expression pET21d derivative vector for C-terminal His_6_-tag fusion, T7 promoter, Amp^R^ | (11) |
| p-*wpsA* | pET21His30 derivative containing *wpsA* from *L. lactis* NZ9000 | This work |
| p-*wpsAB* | pET21His30 derivative containing *wpsA* and *wpsB* from *L. lactis* NZ9000 | This work |
| p-*wpsBAfu* | pET21His30 derivative containing fused *wpsB* and *wpsA* from NZ9000 | This work |
| p-*NZ_wpsC* | pET21His60 derivative containing *wpsC* from NZ9000 | This work |
| pGacIJ | pRSF-NT derivative containing *gacI* and *gacJ* from from *S. pyogenes* MGAS5005 | (12) |
| pMal-c4X | *E. coli* expression vector for N-terminal MBP fusion, *tac* promoter, Amp^R^ | NEB |
| p-SK_WpsC | pMal-c4X derivative containing *wpsC* from *L. lactis* SK11 | This work |
| pNZ8048 | *L. lactis* expression vector, for translational fusion with nisin-inducible *nisA* promoter, Cm^R^ | (13) |
| p-*CD_NZ_* | pNZ8048 derivative containing *wpsC* and *wpsD* genes from *L. lactis* NZ9000 | This work |
| p-*CD_SMQ_* | pNZ8048 derivative containing *wpsC* and *wpsD* genes from *L. lactis* SMQ-388 | This work |
| p-*EF_NZ_* | pNZ8048 derivative containing *wpsE* and *wpsF* genes from *L. lactis* NZ9000 | This work |
| p-*EF_SMQ_* | pNZ8048 derivative containing *wpsE* and *wpsF* genes from *L. lactis* SMQ-388 | This work |
|  |  |  |
| **Bacteriophages** |  |  |
| sk1 | Skunaviridae, propagated on NZ9000 | (14) |
| p2 | Skunaviridae, propagated on NZ9000 | (15) |

**Table S2. Primers used in this study**

| **Primer name** | **Sequence (5’- 3’)^a, b, c,d^** |
| --- | --- |
| **Primers used for the construction of overexpression plasmids** | |
| 218_His30-f | CGGGATCCAAAATTTTACTAATTATCCCAG |
| 218_His30-r | CCGCTCGAGTTACTTACTCTCCTTTCCTAA |
| 219_His30-r | CCGCTCGAGTTAATTTTCCTTATCTTTATCG |
| fuBA-H30B-f | **ATCACCATCACCATCACGGA**CCTATTCAACTTCGTATCTTAG |
| fuBA-B-r | GCTGGGATAATTAGTAAAATTTTTCCATTTTCCTTATCTTTATCGG |
| fuBA-A-f | CCGATAAAGATAAGGAAAATGGAAAAATTTTACTAATTATCCCAGC |
| fuBA-H30A-r | **GGTGGTGGTGGTGGTGCTCGAG**TTACTTACTCTCCTTTCCTA |
| NZ_wpsC-f | CATGCCATGGGAAATAAAGTCATCAAACATACTTT |
| NZ_wpsC-r | GAAGATCTTGAATTTCCTTTTTGATTATTAG |
| Sk_wpsC-f | *PO_4_*-AAAACAGTTGCTATTTTTAG |
| Sk_wpsC-r | GGAATTCTTAATTGATTTCAATTTTTTCTAA |
|  |  |
| **Primers used for the construction of complementation plasmids (cloning in pNZ8048)** | |
| NZ1145-f | **AATTATAAGGAGGCACTCACC**ATGAATAAAGTCATCAAACATACTTTCGT |
| NZ1150-r | **AGTGGTACCGCATGCCTGCA**TCATCCATTGTTAACTCCCAAAAC |
| NZ1155-f | **aattataaggaggcactcacc**ATGGATGAAAAAATTGACTTTATAGTAAC |
| NZ1160-r | **agtggtaccgcatgcctgca**TTATTTATTTTCCTCAATGACTGTT |
| SMQ17-f | **AATTATAAGGAGGCACTCACC**ATGAATAAAATATTGACAATTACAGTGCC |
| SMQ18-r | **AGTGGTACCGCATGCCTGCA**TTATTCATTACCAGACTCTGTTC |
| SMQ19-f | **AATTATAAGGAGGCACTCACC**ATGAATAAAACTTTAGAAAAAATTGATTTTGT |
| SMQ20-r | **AGTGGTACCGCATGCCTGCA**TTATTTATTCTCCTCAAGAACTTTA |
|  |  |
| **Primers used for the validation of recombinant plasmids** | |
| pET21-f | TTAATACGACTCACTATAGGGG |
| pET21-r | TAGTTATTGCTCAGCGGTG |
| pMalE | TCAGACTGTCGATGAAGC |
| pMal-rev | GCGATTAAGTTGGGTAACG |
| msp3545-f | ACGGCTCTGATTAAATTCTG |
| msp3545-f | GCTTGAAACGTTCAATTG |
|  |  |

^a^ Restriction site are underlined.

^b^ Overlap between sequences to assemble amplified *wpsB* and *wpsA* gene into pET21His30 are overlaid in gray.

^c^ Sequences overlapping with vectors are written in bold.

^d^ *PO_4_* indicate that the 5’-end of Sk_wpsC-f was phosphorylated.

**References**

1. Chapot-Chartier, M. P., Vinogradov, E., Sadovskaya, I., Andre, G., Mistou, M. Y., Trieu-Cuot, P., Furlan, S., Bidnenko, E., Courtin, P., Pechoux, C., Hols, P., Dufrene, Y. F., and Kulakauskas, S. (2010) The cell surface of *Lactococcus lactis* is covered by a protective polysaccharide pellicle. *J Biol Chem* **285**, 10464-10471

2. Theodorou, I., Courtin, P., Palussiere, S., Kulakauskas, S., Bidnenko, E., Pechoux, C., Fenaille, F., Penno, C., Mahony, J., van Sinderen, D., and Chapot-Chartier, M. P. (2019) A dual-chain assembly pathway generates the high structural diversity of cell-wall polysaccharides in *Lactococcus lactis*. *J Biol Chem* **294**, 17612-17625

3. Theodorou, I., Courtin, P., Sadovskaya, I., Palussiere, S., Fenaille, F., Mahony, J., Chapot-Chartier, M. P., and van Sinderen, D. (2020) Three distinct glycosylation pathways are involved in the decoration of *Lactococcus lactis* cell wall glycopolymers. *J Biol Chem* **295**, 5519-5532

4. Rismondo, J., Percy, M. G., and Grundling, A. (2018) Discovery of genes required for lipoteichoic acid glycosylation predicts two distinct mechanisms for wall teichoic acid glycosylation. *J Biol Chem* **293**, 3293-3306

5. Rismondo, J., Haddad, T. F. M., Shen, Y., Loessner, M. J., and Grundling, A. (2020) GtcA is required for LTA glycosylation in *Listeria monocytogenes* serovar 1/2a and *Bacillus subtilis*. *Cell Surf* **6**, 100038

6. Ardiccioni, C., Clarke, O. B., Tomasek, D., Issa, H. A., von Alpen, D. C., Pond, H. L., Banerjee, S., Rajashankar, K. R., Liu, Q., Guan, Z., Li, C., Kloss, B., Bruni, R., Kloppmann, E., Rost, B., Manzini, M. C., Shapiro, L., and Mancia, F. (2016) Structure of the polyisoprenyl-phosphate glycosyltransferase GtrB and insights into the mechanism of catalysis. *Nat Commun* **7**, 10175

7. Farenc, C., Spinelli, S., Vinogradov, E., Tremblay, D., Blangy, S., Sadovskaya, I., Moineau, S., and Cambillau, C. (2014) Molecular insights on the recognition of a *Lactococcus lactis* cell wall pellicle by the phage 1358 receptor binding protein. *Journal of virology* **88**, 7005-7015

8. Ainsworth, S., Sadovskaya, I., Vinogradov, E., Courtin, P., Guerardel, Y., Mahony, J., Grard, T., Cambillau, C., Chapot-Chartier, M. P., and van Sinderen, D. (2014) Differences in lactococcal cell wall polysaccharide structure are major determining factors in bacteriophage sensitivity. *mBio* **5**, e00880-00814

9. Gasson, M. J. (1983) Plasmid complements of *Streptococcus lactis* NCDO 712 and other lactic streptococci after protoplast-induced curing. *J Bacteriol* **154**, 1-9

10. Kuipers, O. P., de Ruyter P. G. G. A., Kleerebezem M., and de Vos, W. M. (1998) Quorum sensing-controlled gene expression in lactic acid bacteria. *Journal of Biotechnology* **64**, 15-21

11. Barreteau, H., Bouhss, A., Fourgeaud, M., Mainardi, J. L., Touze, T., Gerard, F., Blanot, D., Arthur, M., and Mengin-Lecreulx, D. (2009) Human- and plant-pathogenic Pseudomonas species produce bacteriocins exhibiting colicin M-like hydrolase activity towards peptidoglycan precursors. *J Bacteriol* **191**, 3657-3664

12. Rush, J. S., Edgar, R. J., Deng, P., Chen, J., Zhu, H., van Sorge, N. M., Morris, A. J., Korotkov, K. V., and Korotkova, N. (2017) The molecular mechanism of N-acetylglucosamine side-chain attachment to the Lancefield group A carbohydrate in *Streptococcus pyogenes*. *J Biol Chem* **292**, 19441-19457

13. de Ruyter, P. G., Kuipers, O. P., and de Vos, W. M. (1996) Controlled gene expression systems for *Lactococcus lactis* with the food-grade inducer nisin. *Applied and environmental microbiology* **62**, 3662-3667

14. Chandry, P. S., Moore, S. C., Boyce, J. D., Davidson, B. E., and Hillier, A. J. (1997) Analysis of the DNA sequence, gene expression, origin of replication and modular structure of the *Lactococcus lactis* lytic bacteriophage sk1. *Mol Microbiol* **26**, 49-64

15. Higgins, D. L., Sanozky-Dawes, R. B., and Klaenhammer, T. R. (1988) Restriction and modification activities from *Streptococcus lactis* ME2 are encoded by a self-transmissible plasmid, pTN20, that forms cointegrates during mobilization of lactose-fermenting ability. *J Bacteriol* **170**, 3435-3442
